# Supplementary material for: Controlled X‐chromosome dynamics defines meiotic potential of female mouse in vitro germ cells
Source: EMBO J. 2022 May 23;41(12):e109457. doi: 10.15252/embj.2021109457 (PMC9194795; doi:10.15252/embj.2021109457)
Supplement: Supplementary file 1 — Expanded View Figures PDF [file EMBJ-41-e109457-s005.pdf]

Expanded View Figures

Figure EV1. A tailor-made system to trace X-chromosome inactivation and reactivation dynamics during PGCLC induction.

A The left panel shows representative contour plots of FACS analysis of PGCLC induction, without or with Dox, in PGCLC d4 induced from EpiLC d2. The right panel shows PGCLC d5 induced from EpiLC d4. The number indicates the percentage of gated germ cells identified by CD61 and SSEA1 signal. Shown are contour plots gated on live cells.

B Bar plots showing XGFP percentages from CD61+ SSEA1+ PGCLCs. Each dot represents a separate induction (n = 3), performed in two biological clones.

C Quantitative RT–PCR of XGFP and XTomato reporters throughout the differentiation timeline, normalized to embryonic stem cells. Horizontal line indicates mean fold change for three separate inductions, using two different clones.

D Immunolabelling with antibodies against SOX2 (yellow), XGFP (green) and H3K27me3 (red) in EpiLCs, PGCLCs d1 and PGCLCs d5. Images show representative groups of cells showing H3K27me3 enrichment on the X^{mus}. The white squares represent the position of the magnified region at the right. Cells were counterstained with DAPI (grey). Dashed line indicates SOX2+/H3K27me3- cells. Continuous line indicates SOX2+/H3K27me3+ cells. Scale bar, 50 and 10 µm for the magnified region.

E Barplots indicating the percentage of cells having H3K27me3 accumulation. PGCLC d1 and PGCLC d5 H3K27me3 percentages are calculated from SOX2-positive cells. On top of the bars, the total cell number analysed from n = 3 separate inductions, using two biological clones, is indicated.

F Barplots indicating the percentage of H3K27me3 accumulation separated by XGFP− and XGFP+ cells. PGCLC d1 and PGCLC d5 H3K27me3 percentages are calculated from SOX2-positive cells. On top of the bars, the total cell number analysed from n = 3 separate inductions, using two biological clones is indicated.

© 2022 The Authors

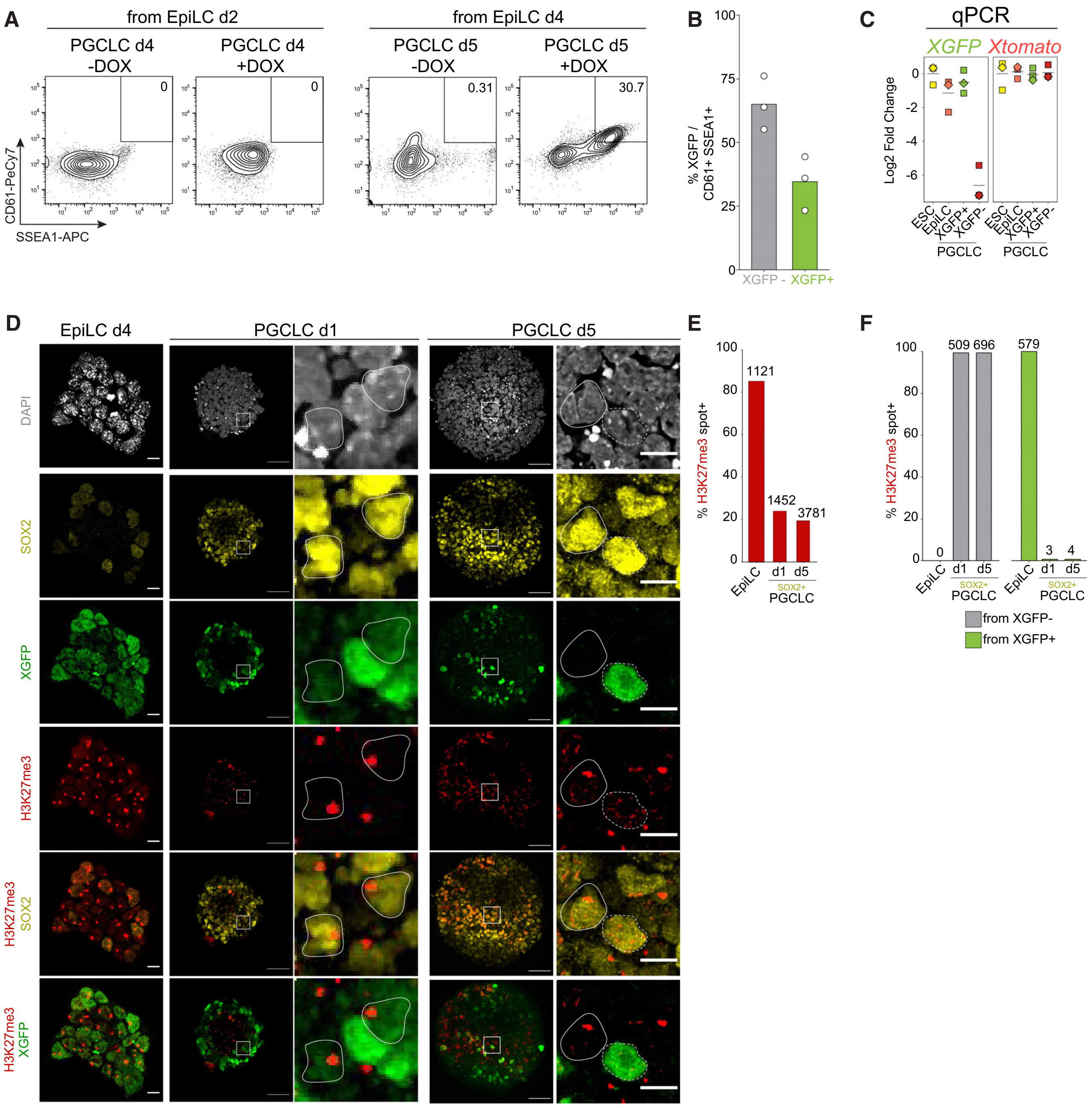
A
from EpiLC d2
from EpiLC d4
PGCLC d4 -DOX
PGCLC d4 +DOX
PGCLC d5 -DOX
PGCLC d5 +DOX
0
0
0.31
30.7
CD61-PeCy7
SSEA1-APC
B
% XGFP / CD61+ SSEA1+
XGFP -
XGFP+
C
qPCR
XGFP
Xtomato
Log2 Fold Change
ESC
EpiLC
XGFP+
XGFP-
PGCLC
D
EpiLC d4
PGCLC d1
PGCLC d5
DAPI
SOX2
XGFP
H3K27me3
E
% H3K27me3 spot+
1121
1452
3781
EpiLC
d1
d5
SOX2+
PGCLC
F
509
696
579
0
3
4
from XGFP-
from XGFP+

Figure EV1.

Figure EV2. Gene expression analysis reveals two PGCLC subpopulations.

A Top panel shows representative contour plots of FACS analysis of PGCLC induction, without or with Dox, in PGCLC d5. The number indicates the percentage of gated germ cells identified by CD61 and SSEA1 signal. Bottom panel indicates the percentages of XGFP+ (green box) or XGFP− (red box) cells. For the +Dox condition, percentages of XGFP+ and XGFP− cells originating from SSEA1+/CD61+ gate are shown. Shown are contour plots gated on live cells.

B PCA of gene expression dynamics during PGCLC differentiation. n = Top 500 most variable genes excluding X-chromosomal genes. Axes indicate the variance. Arrows indicate hypothetical trajectory. Shapes indicate the clones (A11 = square, E9 = rhombus).

C Quantitative RT–PCR of selected differentially expressed early germ cell marker genes in ESCs, EpiLCs, XGFP+ PGCLCs and XGFP− PGCLCs during the differentiation time course. Each point represents a separate biological replicate, originating from either clone A or clone E. n = 3 biological replicates. The numbers above the bars indicate P-values (two-sample unpaired Wilcoxon–Mann–Whitney test with *R* defaults).

D Expression levels (normalized DEseq2 counts) of selected differentially expressed meiosis marker genes in ESCs, EpiLCs, XGFP+ PGCLCs and XGFP− PGCLCs during the differentiation time course. Genes with FDR < 0.001 were considered significantly differentially expressed. Points indicate expression of individual biological replicates.

E Representative images showing the X-activity reporter status in colonies formed by ESCs, XGFP+ PGCLCs and XGFP− PGCLCs after 7 days of culture in 2i/LIF on immortalized mouse embryonic fibroblasts. BF, bright field. Scale bars = 200 μm.

F FACS analysis showing the X-reporter status of the indicated cell types after 7 days of culture in 2i/LIF on immortalized mouse embryonic fibroblasts. Numbers indicate the percentage of cells falling in the corresponding gate. Histograms come from XTomato+ gated cells depleted of immortalized mouse embryonic fibroblasts.

 © 2022 The Authors

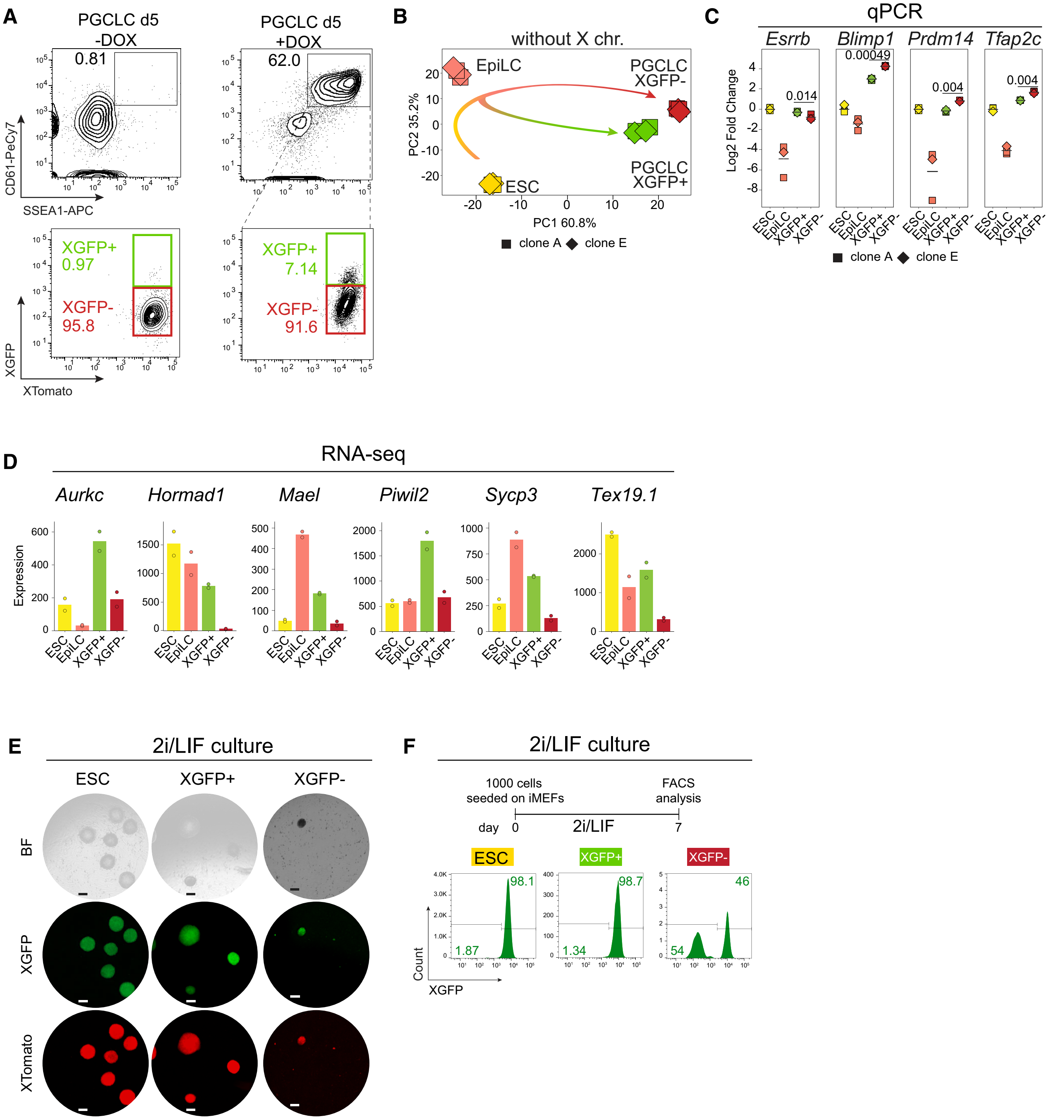
A
PGCLC d5 -DOX
PGCLC d5 +DOX
0.81
62.0
CD61-PeCy7
SSEA1-APC
XGFP+ 0.97
XGFP- 95.8
XGFP+ 7.14
XGFP- 91.6
XGFP
XTomato
B
without X chr.
EpiLC
PGCLC XGFP-
PGCLC XGFP+
ESC
PC2 35.2%
PC1 60.8%
clone A clone E
C
qPCR
Esrrb
Blimp1
Prdm14
Tfap2c
Log2 Fold Change
0.014
0.00049
0.004
0.004
ESC EpiLC XGFP+ XGFP-
clone A clone E
D
RNA-seq
Aurkc
Hormad1
Mael
Piwil2
Sycp3
Tex19.1
Expression
ESC EpiLC XGFP+ XGFP-
E
2i/LIF culture
ESC
XGFP+
XGFP-
BF
XGFP
XTomato
F
2i/LIF culture
1000 cells seeded on iMEFs
FACS analysis
day 0 2i/LIF 7
ESC
XGFP+
XGFP-
98.1
1.87
98.7
1.34
46
54
Count
XGFP

Figure EV2.

Figure EV3. Characterization of X-inactivation dynamics during PGCLC induction.

A Distribution of the allelic ratio of X-linked genes in ESCs. Dashed lines indicate a biallelic expression window from 0.3 to 0.7. *n* = 334 genes.
B Distribution of the allelic ratio in NPCs of X-linked genes expressed biallelically in ESCs. Dashed line represents allelic ratio of 0.135 used as a threshold for X-inactivation. Genes below the threshold are considered X-inactive. *n* = 294 genes.
C Boxplots of allelic ratio of genes located on chromosome 13 (*n* = 294). Dashed line indicates the biallelic ratio of 0.5. Box plots depict the first and third quartiles as the lower and upper bounds of the box, with a band inside the box showing the median value and whiskers representing 1.5x the interquartile range.
D Expression of X^{mus} genes in ESCs belonging to the indicated categories. The numbers above the bars indicate *P*-values (two-sample unpaired Wilcoxon–Mann–Whitney test with *R* defaults). Box plots depict the first and third quartiles as the lower and upper bounds of the box, with a band inside the box showing the median value and whiskers representing 1.5x the interquartile range. *n* = 295 genes.
E Expression of X^{mus} genes in XGFP− PGCLCs belonging to the indicated categories. The numbers above the bars indicate *P*-values (two-sample unpaired Wilcoxon–Mann–Whitney test with *R* defaults). Box plots depict the first and third quartiles as the lower and upper bounds of the box, with a band inside the box showing the median value and whiskers representing 1.5x the interquartile range. *n* = 295 genes.
F Venn diagram of the overlap between X-inactivation gene groups in this study with the early, late and escapee genes during embryoid body (EB) differentiation (Marks *et al*, 2015)
G Marker gene expression projected onto the UMAP plot of Fig 3G.
H Violin plots showing the pluripotency score of E6.5 clusters calculated using the expression of *Pou5f1*, *Sox2*, *Nanog*, *Klf2*, *Esrrb*, *Dppa3*, *Tfcp2l1* and *Prdm14*. Dots represent single cells. The numbers above indicate *P*-values (two-sample unpaired Wilcoxon–Mann–Whitney test with *R* defaults).

 © 2022 The Authors

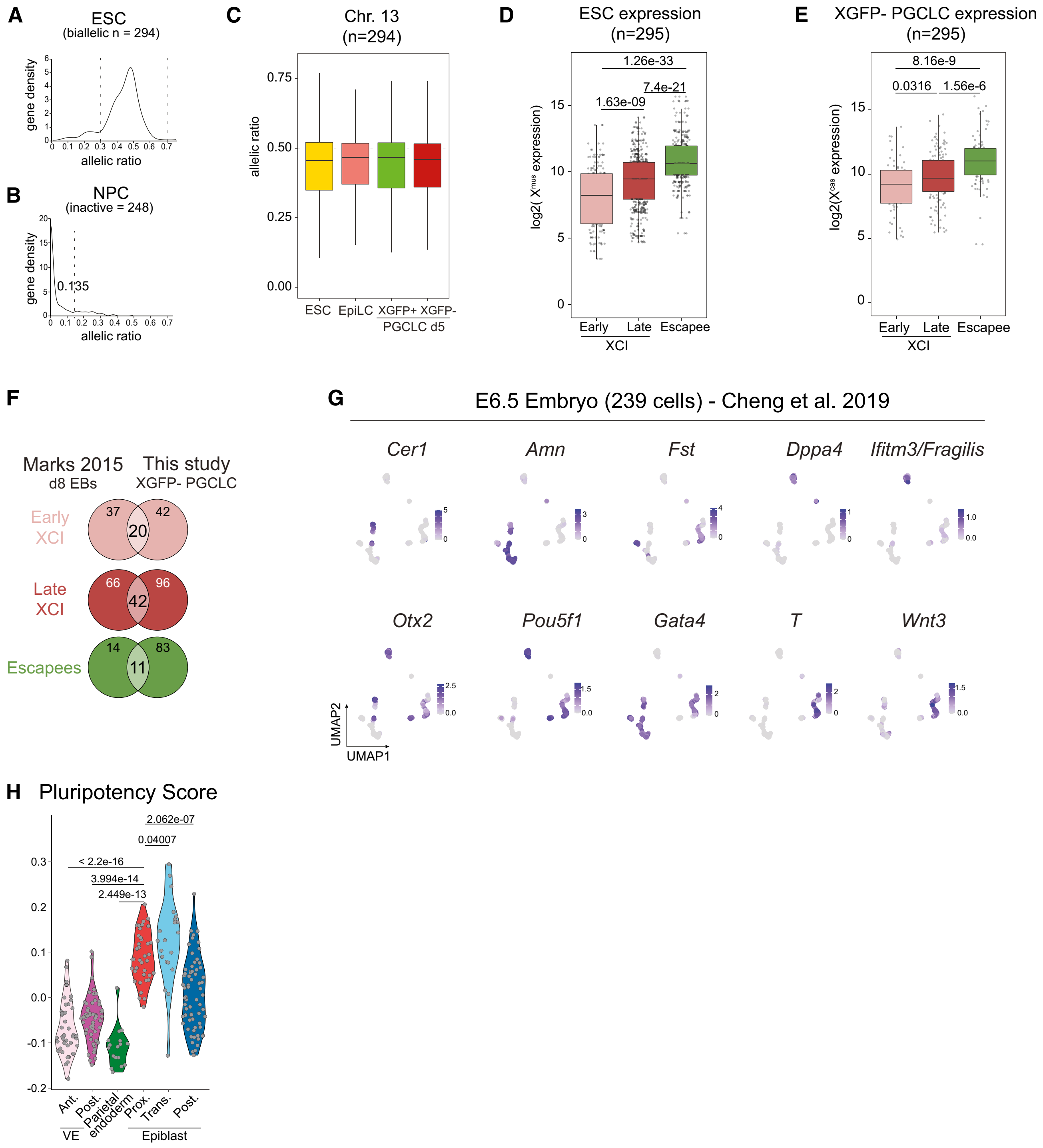
A
ESC
(biallelic n = 294)
gene density
allelic ratio
B
NPC
(inactive = 248)
0.135
gene density
allelic ratio
C
Chr. 13
(n=294)
allelic ratio
0.75
0.50
0.25
0.00
ESC
EpiLC
XGFP+
XGFP-
PGCLC d5
D
ESC expression
(n=295)
1.26e-33
7.4e-21
1.63e-09
log2(Xmus expression)
Early
Late
Escapee
XCI
E
XGFP- PGCLC expression
(n=295)
8.16e-9
0.0316
1.56e-6
log2(Xcas expression)
Early
Late
Escapee
XCI
F
Marks 2015
d8 EBs
This study
XGFP- PGCLC
Early XCI
37
20
42
Late XCI
66
42
96
Escapees
14
11
83
G
E6.5 Embryo (239 cells) - Cheng et al. 2019
Cer1
Amn
Fst
Dppa4
Ifitm3/Fragilis
Otx2
Pou5f1
Gata4
T
Wnt3
UMAP2
UMAP1
H
Pluripotency Score
2.062e-07
0.04007
< 2.2e-16
3.994e-14
2.449e-13
Ant.
Post.
Parietal endoderm
Prox.
Trans.
Post.
VE
Epiblast

Figure EV3.

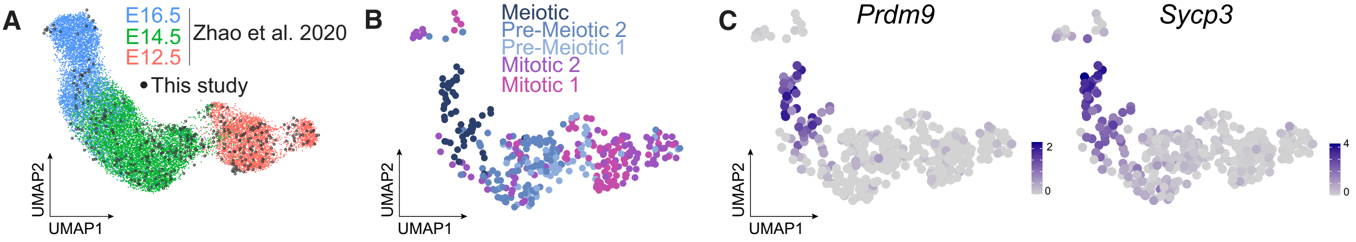

Figure EV4. Single-cell RNA-seq of maturing germ cells using the rOvary system in comparison to *in vivo* data.

A Integration with *in vivo* published single-cell RNA-seq data from E12.5 (red), E14.5 (green) and E16.5 (blue) (Zhao *et al*, 2020). Black dots represent cells from *in vitro* rOvaries from this study.

B Distribution of germ cell maturation clusters from rOvaries identified in this study, along the *in vivo* UMAP projection.

C Meiotic marker gene expression of germ cell maturation clusters from rOvaries identified in this study, projected onto the in *vivo* UMAP plot. Cells on top left of the plot (in B and C), despite falling into the E16.5 cluster, represent deviant cells of abnormal character by not showing expression of late meiotic markers *Prdm9* and *Sycp3*.

© 2022 The Authors
