# Supplementary figures and images for: Controlled X‐chromosome dynamics defines meiotic potential of female mouse in vitro germ cells
Source: EMBO J. 2022 May 23;41(12):e109457. doi: 10.15252/embj.2021109457 (PMC9194795; doi:10.15252/embj.2021109457)

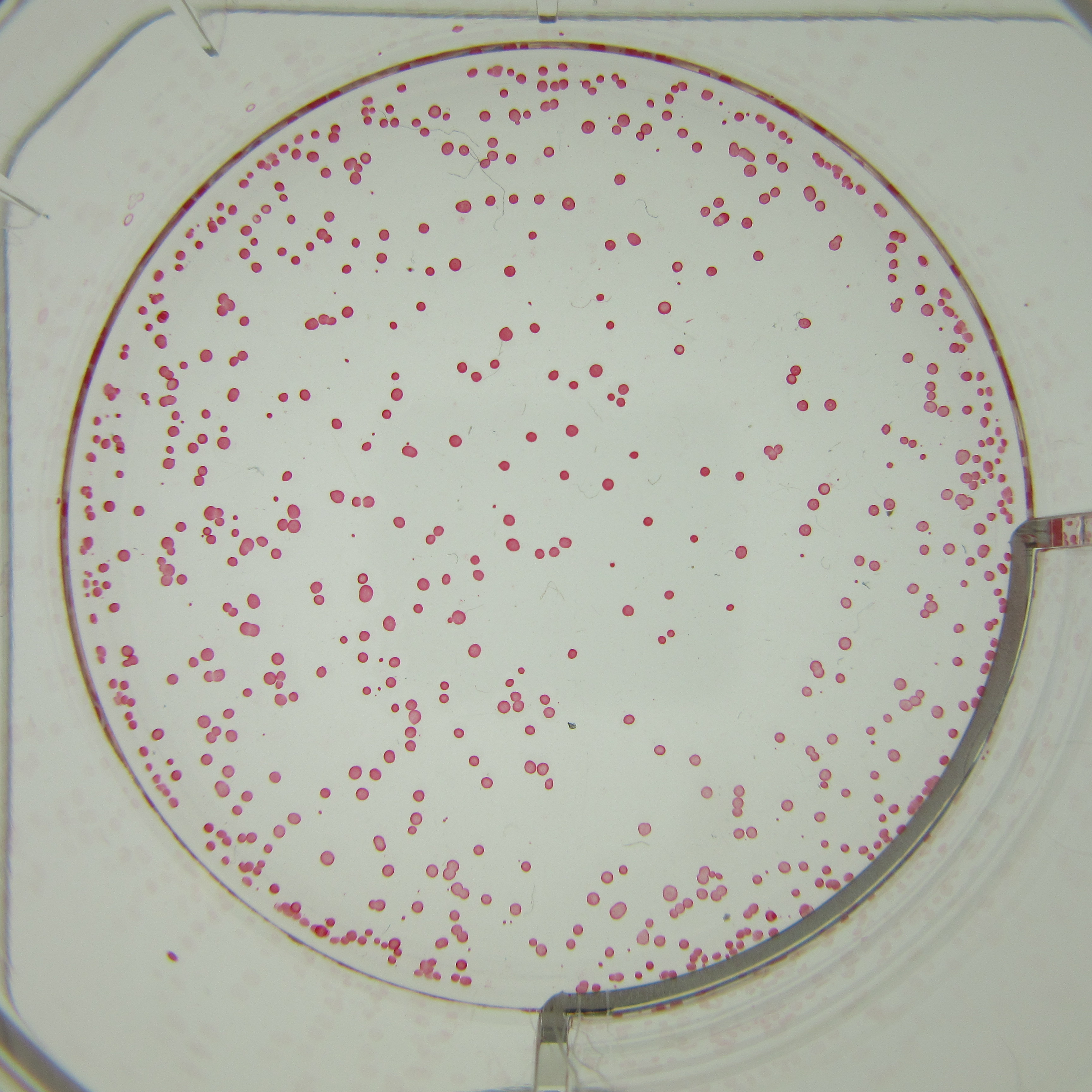

Supplement: Supplementary file 6 — Source Data for Figure 2 [file EMBJ-41-e109457-s002.zip › Fig2H_ESC.JPG]

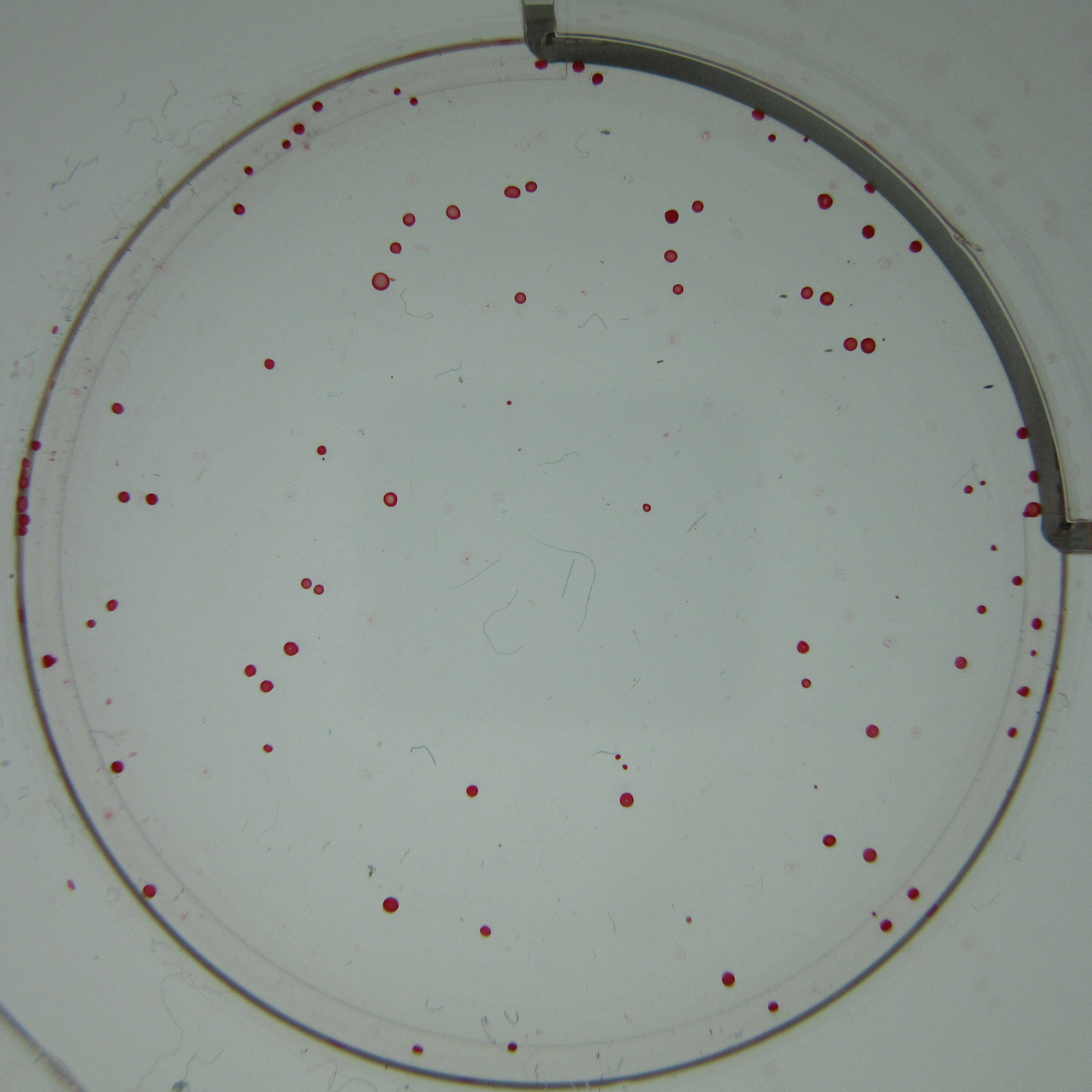

Supplement: Supplementary file 6 — Source Data for Figure 2 [file EMBJ-41-e109457-s002.zip › Fig2H_XGFP+.JPG]

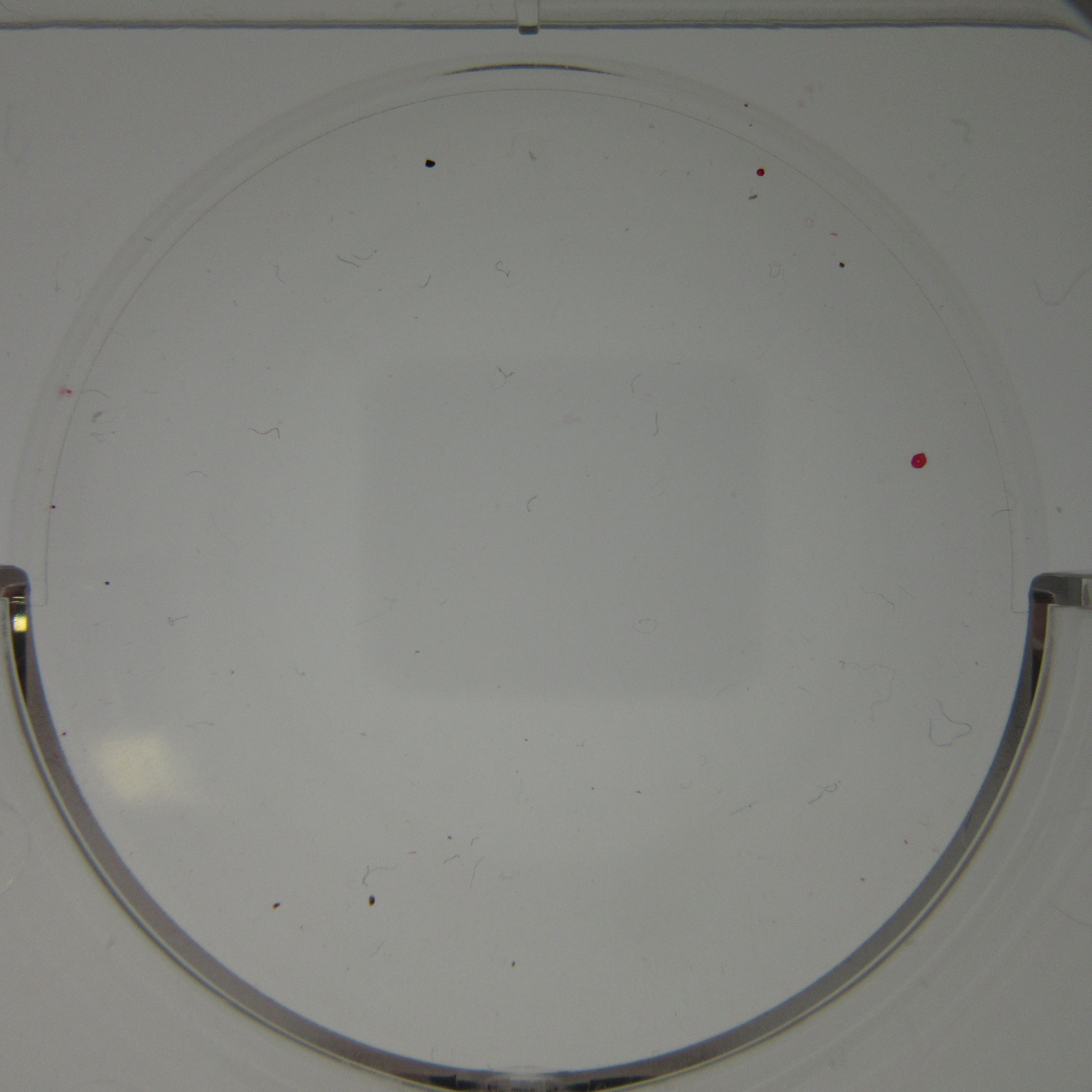

Supplement: Supplementary file 6 — Source Data for Figure 2 [file EMBJ-41-e109457-s002.zip › Fig2H_XGFP-.JPG]
