# Supplementary material for: Controlled X‐chromosome dynamics defines meiotic potential of female mouse in vitro germ cells
Source: EMBO J. 2022 May 23;41(12):e109457. doi: 10.15252/embj.2021109457 (PMC9194795; doi:10.15252/embj.2021109457)
Supplement: Supplementary file 7 [file EMBJ-41-e109457-s007.pdf]

THE EMBO JOURNAL

Controlled X-chromosome dynamics defines meiotic potential of female mouse in vitro germ cells

Jacqueline Serverino, Moritz Bauer, Tom Mattimoe, Niccolo Arecco, Luca Cozzuto, Patricia Lorden, Norio Hamada, Yoshiaki Nosaka, So I. Nagaoka, Pauline Audergon, Antonio Tarruell, Holger Heyn, Katsuhiko Hayashi, Mitinori Saitou, and Bernhard Payer
DOI: 10.15252/embj.2021109457

Corresponding author: Bernhard Payer (bernhard.payer@crg.eu)

Review Timeline:

Submission Date:	16th Aug 21
Editorial Decision:	13th Sep 21
Revision Received:	22nd Feb 22
Editorial Decision:	27th Mar 22
Revision Received:	8th Apr 22
Accepted:	14th Apr 22

Editor: Daniel Klimmeck

Transaction Report:

(Note: With the exception of the correction of typographical or spelling errors that could be a source of ambiguity, letters and reports are not edited. Depending on transfer agreements, referee reports obtained elsewhere may or may not be included in this compilation. Referee reports are anonymous unless the Referee chooses to sign their reports.)

Dear Dr Payer,

Thank you again for the submission of your manuscript (EMBOJ-2021-109457) to The EMBO Journal. Please accept my apologies for the unusual delay with the peer-review of your work due to protracted referee input and detailed discussions in the team. Your study has been sent to three referees, and we have received reports from all of them, which I enclose below.

As you will see, the referees acknowledge the potential interest of your model and value of the time-resolved results in a timely context, although they also express major concerns, which need to be addressed before they can be supportive of publication at the EMBO Journal. In more detail, the referees state substantial issues regarding the sensitivity of the in vitro system and in vivo relevance of the findings (referees #1 and #2). Further, reviewer #3 states that the functional relevance of the linkage between X inactivation status and differentiation capacity is unclear, which in his-her view weakens the impact of the results. Finally, all three referees list technical issues and controls related to the data presented and methods annotation, that would need to be addressed to achieve the level of robustness needed for The EMBO Journal.

Given the referees' positive recommendations and detailed constructive comments, I would like to invite you to submit a revised version of the manuscript, addressing the issues raised. I should add that it is EMBO Journal policy to allow only a single round of revision, and acceptance of your manuscript will therefore depend on the completeness of your responses in this revised version.

In light of the extensive experimentation requested by the reviewers, I would appreciate if you could contact me during the next weeks via e.g. a video call to discuss your perspective on the comments and potential plan for revisions.

We generally allow three months as standard revision time. As a matter of policy, competing manuscripts published during this period will not negatively impact on our assessment of the conceptual advance presented by your study. However, we request that you contact the editor as soon as possible upon publication of any related work, to discuss how to proceed. Should you foresee a problem in meeting this three-month deadline, please let us know in advance and we may be able to grant an extension.

When submitting your revised manuscript, please carefully review the instructions below.

Thank you for the opportunity to consider your work for publication.
I look forward to your revision.

Kind regards,

Daniel Klimmeck

Daniel Klimmeck, PhD
Senior Editor
The EMBO Journal

Instruction for the preparation of your revised manuscript:

1) a .docx formatted version of the manuscript text (including legends for main figures, EV figures and tables). Please make sure that the changes are highlighted to be clearly visible.

2) individual production quality figure files as .eps, .tif, .jpg (one file per figure).

3) a .docx formatted letter INCLUDING the reviewers' reports and your detailed point-by-point response to their comments. As part of the EMBO Press transparent editorial process, the point-by-point response is part of the Review Process File (RPF), which will be published alongside your paper.

4) a complete author checklist, which you can download from our author guidelines (https://wol-prod-cdn.literatumonline.com/pb-assets/embo-site/Author Checklist%20-%20EMBO%20J-1561436015657.xlsx). Please insert information in the checklist that is also reflected in the manuscript. The completed author checklist will also be part of the RPF.

5) Please note that all corresponding authors are required to supply an ORCID ID for their name upon submission of a revised manuscript.

6) It is mandatory to include a 'Data Availability' section after the Materials and Methods. Before submitting your revision, primary datasets produced in this study need to be deposited in an appropriate public database, and the accession numbers and database listed under 'Data Availability'. Please remember to provide a reviewer password if the datasets are not yet public (see https://www.embopress.org/page/journal/14602075/authorguide#datadeposition).
In case you have no data that requires deposition in a public database, please state so in this section. Note that the Data Availability Section is restricted to new primary data that are part of this study.
*** Note - All links should resolve to a page where the data can be accessed. ***

7) Our journal encourages inclusion of *data citations in the reference list* to directly cite datasets that were re-used and obtained from public databases. Data citations in the article text are distinct from normal bibliographical citations and should directly link to the database records from which the data can be accessed. In the main text, data citations are formatted as follows: "Data ref: Smith et al, 2001" or "Data ref: NCBI Sequence Read Archive PRJNA342805, 2017". In the Reference list, data citations must be labeled with "[DATASET]". A data reference must provide the database name, accession number/identifiers and a resolvable link to the landing page from which the data can be accessed at the end of the reference. Further instructions are available at .

8) We would also encourage you to include the source data for figure panels that show essential data. Numerical data can be provided as individual .xls or .csv files (including a tab describing the data). For 'blots' or microscopy, uncropped images should be submitted (using a zip archive or a single pdf per main figure if multiple images need to be supplied for one panel). Additional information on source data and instruction on how to label the files are available at .

9) We replaced Supplementary Information with Expanded View (EV) Figures and Tables that are collapsible/expandable online (see examples in https://www.embopress.org/doi/10.15252/embj.201695874). A maximum of 5 EV Figures can be typeset. EV Figures should be cited as 'Figure EV1, Figure EV2" etc. in the text and their respective legends should be included in the main text after the legends of regular figures.

- For the figures that you do NOT wish to display as Expanded View figures, they should be bundled together with their legends in a single PDF file called *Appendix*, which should start with a short Table of Content. Appendix figures should be referred to in the main text as: "Appendix Figure S1, Appendix Figure S2" etc. See detailed instructions regarding expanded view here: .

- Additional Tables/Datasets should be labelled and referred to as Table EV1, Dataset EV1, etc. Legends have to be provided in a separate tab in case of .xls files. Alternatively, the legend can be supplied as a separate text file (README) and zipped together with the Table/Dataset file.

10) When assembling figures, please refer to our figure preparation guideline in order to ensure proper formatting and readability in print as well as on screen:
http://bit.ly/EMBOPressFigurePreparationGuideline

Please remember: Digital image enhancement is acceptable practice, as long as it accurately represents the original data and conforms to community standards. If a figure has been subjected to significant electronic manipulation, this must be noted in the figure legend or in the 'Materials and Methods' section. The editors reserve the right to request original versions of figures and the original images that were used to assemble the figure.

11) For data quantification: please specify the name of the statistical test used to generate error bars and P values, the number (n) of independent experiments (specify technical or biological replicates) underlying each data point and the test used to calculate p-values in each figure legend. The figure legends should contain a basic description of n, P and the test applied. Graphs must include a description of the bars and the error bars (s.d., s.e.m.).

Further information is available in our Guide to Authors: https://www.embopress.org/page/journal/14602075/authorguide

Revision to The EMBO Journal should be submitted online within 90 days, unless an extension has been requested and approved by the editor; please click on the link below to submit the revision online before 12th Dec 2021:

Link Not Available

Referee #1:

The paper by Severino et al. examines the dynamic of X chromosome inactivation and reactivation during in vitro generation of mouse primordial germ cells in vitro. The authors establish an elegant system to look at the XCI status in female embryonic stem cells being induced towards the germline lineage. This system is used in multiple culture systems followed by bulk and single-cell sequencing. Importantly, the authors conclude that the initiation of the germline lineage is uncoupled from the XCI however successful germline development requires XCI. Furthermore, X chromosome reactivation occurs in all cells, which embark on meiotic division.

The strength of this study is the that the authors have performed elegant time-resolved characterisation of transcriptional inactivation/reactivation of the X chromosome of a well-studied in vitro PGCLC system. By using a hybrid reporter line they were not only able to characterise the Xi status but also uncover the functional importance of XCI in germline development. The main limitation of this report is also related to the model used. In vitro PGCLC generation likely progresses through a different dynamics than in vivo. In particular XCI seems slower in EpiLCs than in vivo as only partial gene silencing is observed after 4days in culture, while in vivo within 2 days of implantation most cells have silenced the X (Cheng et al., 2019). Secondly, the X-GFP reporter system is not very sensitive to changes in the XCI levels. The authors manage to reach important conclusions despite these limitations, however fail to acknowledge them properly. The authors need to amend this and significantly change the flow of the paper. Moreover some important control experiments are also missing. After addressing these concerns I think this paper will be of great importance to the field of epigenetics and X inactivation.

Major points:

1) Results from Figure 4 H on its own are very confusing and counterintuitive. In fact they show that the X-GFP system is not suitable for measuring the levels of XCR. I suggest the following: rename the "GFP-" population to "GFP low" as on FACS there is still some signal. Immediately after this section describe the results now in Fig 6. It is important to show that in fact XCR takes place. This is also the moment when the major limitation of this system becomes apparent and should be appropriately acknowledged.
2) Replicates are missing throughout the manuscript. Most IF/FACS analysis were performed only once. This has to be amended and statistics included whenever possible.
3) Page 6. Conclusions from Fig EV1C are very convoluted and experiments are missing. Are there H3K27me3 foci in GFP+ve Sox2+ve cells? This staining needs to be performed. Can this be due to a high protein stability of GFP rather than the transcriptional status of the locus? This is an important limitation of this model and should be clearly addressed. It cannot be stated that H3K7me3 precedes gene silencing based on Zylicz et al 2019, as gene silencing at some loci is extremely rapid and quicker than H3K27me3 accumulation. Finally it is unclear what the first bar is in Fig ev1C.
4) The allelic ratio of 0.135 seems rather arbitrary. Are the conclusions holding when other thresholds are used? The degree of escape in Figure 3 is very high, how does that relate to findings from in vivo E6.5 epiblast? Published data should be used to check if this is not an in vitro artefact. Finally, how does the early, late and escapee list of genes relate to published datasets?
5) As it is, the discussion does not address key limitations of this system. It should be properly explained and discussed. Comparison to what happens and what could be different in vivo should also be discussed.

Minor points:
1) Line221-224: PC2 differences are never interpreted.
2) Figure 4 G: the dashed cells should be discussed in the text.
3) From the text it remains unclear for me why the authors had to do the experiments in Fig 5. I'm guessing that they allow more robust meiotic activation but this should be properly explained in the text.
4) Figure 2D: the levels of Prdm14, Tfap2c, Blimp1, Esrrb are very similar between the GFP+ve and -ve cells. Such tiny differences should be validated using qPCR but with more replicates.

Referee #2:

Severino, Bauer and colleagues use an in vitro system of inducing germ cell fate and initial oogenesis from mouse embryonic stem cells to study the dynamics of X-inactivation/reactivation. Their hybrid cell line, harbouring biased X-inactivation and X-linked fluorescent reporters allows them to follow X-inactivation in this system with unprecedented detail.

I disagree with the authors that their experiments shows that X-inactivation dynamics impacts germ cell maturation and its potential to entry into meiosis (see below). I believe the major contribution of this manuscript is the high resolution, detailed view on the timing of X-inactivation & reactivation in the development of female germ cells. The work has its merits given the difficulties to perform these experiments in vivo, and the findings should be highlighted/discussed despite and in light of the shortcomings of the artificial in vitro system. I believe the work has significant appeal to the fields of X-inactivation and germline developmental biology, and I support the publication after minor experimental revision (see point 2) and major changes on the statements and discussion or additional experimental support for the claim that X-inactivation/reactivation may impact meiotic potential (see point 1).

1. (First and second result sessions, lines 270 and 463-465) Claim that X-inactivation may be essential for proper PGCLC maturation and entry into meiosis:

Based on the presented experiments, the absence of XGFP+ in the pre-meiotic and meiotic populations obtained in vitro cannot be attributed to their failure in X-inactivation. The causal relationship between failure to inactivate X(mus) and improper maturation/meiosis progression has not been really tested. It is equally possible (and supported by their data on Figure 2) that XGFP+ cells represent an aberrant PGCLC population in which the failure to undergo X-inactivation is yet merely another manifestation of their aberrant state.

A percentage of aberrant PGCLCs may be an unsurmountable shortcoming of the in vitro PGCLC system, or it may be that aberrant reprogramming also occurs in vivo. Perhaps the massive attrition of foetal oocytes, and the somewhat less extensive attrition of gonocytes observed in vivo reflects this phenomenon. I think the definite experiment would involve testing if the implantation of blastocysts made chimeric with Xist KO ES cells could lead to females in which the ES cells contributes to oogonia at the meiotic prophase I. However, this seems like an unreasonable request for the scope of this manuscript.

Perhaps a more feasible experiment would be to produce a Xist KO (or X-inactivation deficient) ES cell line on top of their XRep, follow with the EpiLC-PGCLC differentiation, and use the entirety of SSEA1/CD61 cells-positive cells in the rOvary reconstitution. Since in Xist WT XRep cells the XGFP- (meiotic competent) reliably constitute 60% of their PGCLC population, the number of cells that successfully enter meiosis should roughly follow the 60/40% of XGFP-/XGFP+. Despite the challenges of making such an experiment quantitative to an extent where these proportions can be followed throughout the in vitro differentiation protocols, it would at least provide some evidence that X-inactivation is required for PGCLC development/maturation.

In any case, I think it would be fair to discuss these potential shortcomings more openly. Instead, the authors attempt to sell the system as more advantageous, and that their data supports a model where X-chromosome dosage compensation kinetics and germline development are tied under a functional link (lines 613-628).

2. Related to the shortcomings of the in vitro PGCLC system for studying X-inactivation: what is the percentage of XGFP- and XGFP+ cells at the day 4 of EpiLCs? Based on Figure 1F it seems that X-inactivation has barely started at the point where cell fate is pushed towards PGCLCs (although it seems the figure shows only the percentage of gated PGC-marker positive, SSEA1/CD61 cells, which should not be present anyway at this point). It would be important to show this data for the entirety of EpiLCs in comparison and discuss how does this compare to the dynamics of X-inactivation in vivo, at the stage when PGCs are specified.

Referee #3:

The manuscript characterizes the X inactivation and reactivation dynamics during in vitro differentiation of female germ cells. To this end, a female mouse embryonic stem cell line (XRep) is used that allows allele-specific monitoring of X inactivation by allele-specific RNA-seq and by flow cytometry through X-linked fluorescent reporters. The manuscript reports the dynamics of X inactivation and X reactivation during differentiation of the XRep cell line towards female germ cells, assessed by bulk and single-cell RNA-seq. To understand how the X inactivation status of a cell correlates with its differentiation potential, the X-linked fluorescent reporters are used to sort cells according to their X inactivation status after PGCLC differentiation. Cells that have not yet undergone X inactivation are found to be less differentiated and to not be able to progress towards meiosis. While this is an interesting observation, it is not clear how it should be interpreted. As also pointed out in the discussion section, it is unclear whether there is a functional link between X inactivation status and differentiation potential or whether the two processes are just temporally correlated. Moreover, I am not sure how the specific parameters of the in vitro differentiation systems (e.g. length of culture in a specific condition) affect the result and to what extent they are thus indicative of the process in vivo.

Major points

1. When assessing GFP expression during the differentiation procedure, it looks like EpiLCs at day 4 are just initiating X inactivation (Fig. 1F, 3B). Since these cells in vivo have already undergone X inactivation, the in vitro system used does not seem to recapitulate this correctly. Maybe the EpiLC culture should be extended or the differentiation protocol should be optimized?
2. When the XGFP+ cells are sorted at day 5 of PGCLC differentiation, these represent 7% of cells that appear to be slightly delayed in differentiation compared to the XGFP- cells that have undergone X inactivation (Fig. 2, EV2). In the initial characterization of the system in Fig. 1F, 40% of cells are XGFP+ at day 5, suggesting less efficient differentiation in that experiment. Would then a longer differentiation towards PGCLC allow full X inactivation and thus more efficient differentiation towards the germ line?
3. The manuscript suggests that the xGFP+ cells, which later exhibited impaired differentiation, might "correspond to an aberrant PGCLC state with similarities to ESCs" (p. 10, line 250). I don't understand how this conclusion is reached, since the presented data rather suggests that the differentiation speed is heterogeneous and that these cells are somewhat delayed in their differentiation, and might actually be able to further differentiate, if cultured under PGCLC conditions for longer?

4. In Fig. 5 cells are differentiated beyond the PGCLC state, which leads to X reactivation in XGFP- cells. It would be useful to see this test for XGFP+ cells as well to support the idea that X inactivation is required for germ cell specification.

Minor points

5. I find the color scale used for the heatmaps misleading (Fig. 3C, 6A, 6C) as it is centered around 0.13. I would suggest to use a two-color system centered around 0.5, as this corresponds of biallelic expression.
6. Although the fluorescent reporters are a useful tool to assess X inactivation, their (binary) activity cannot be used to discern whether a cell has undergone X inactivation or not. Since X inactivation as well as X reactivation occurs with variable dynamics across genes, it can be misleading to describe it as a binary process. On p. 6, line 153 for example it is stated that "XGFP+ PGCLCs failed to undergo X-linked gene silencing...". In such instances it would be more precise to state that cells failed to undergo silencing of the X-linked GFP transgene.
7. According to the legend Fig. 1F shows all live cells, but the XGFP percentages is only calculated within SSEA1+/CD61+ cells. Is this correct? I would suggest to calculate the percentages for the cells shown in the plot.

Other points

8. I would suggest to invert the order of Fig. 5 and Fig. 6, since the characterization of XCI by allele-specific scRNA-seq analysis in Fig. 6 follows logically from Fig. 4H, which looks at the X inactivation status in the same data set by flow cytometry.
9. The font size of the labels in some of the heatmaps are too small.

Point by Point Response to Reviewers Questions for Severino et al. “Distinct X-chromosome dynamics characterizes female mouse in vitro germ cells with meiotic potential” (EMBOJ-2021-109457R)

AUTHOR'S RESPONSE: We thank all reviewers for their positive and insightful comments on our manuscript, and greatly appreciate their help and guidance for improving it during the revision process. Below we address all the points raised and highlight in yellow new additions/modifications to the manuscript text.

Referee #1:

The paper by Severino et al. examines the dynamic of X chromosome inactivation and reactivation during in vitro generation of mouse primordial germ cells in vitro. The authors establish an elegant system to look at the XCI status in female embryonic stem cells being induced towards the germline lineage. This system is used in multiple culture systems followed by bulk and single-cell sequencing. Importantly, the authors conclude that the initiation of the germline lineage is uncoupled from the XCI however successful germline development requires XCI. Furthermore, X chromosome reactivation occurs in all cells, which embark on meiotic division.

The strength of this study is the that the authors have performed elegant time-resolved characterisation of transcriptional inactivation/reactivation of the X chromosome of a well-studied in vitro PGCLC system. By using a hybrid reporter line they were not only able to characterise the Xi status but also uncover the functional importance of XCI in germline development. The main limitation of this report is also related to the model used. In vitro PGCLC generation likely progresses through a different dynamics than in vivo. In particular XCI seems slower in EpiLCs than in vivo as only partial gene silencing is observed after 4days in culture, while in vivo within 2 days of implantation most cells have silenced the X (Cheng et al., 2019).

AUTHOR'S RESPONSE: We thank the reviewer for the positive assessment of our *in vitro* PGCLC differentiation experiments and agree on the potential limitations that the *in vitro* system poses when used in isolation. Therefore we have now newly added an analysis of the X-chromosome dynamics during early mouse postimplantation development *in vivo* (data obtained from (Cheng *et al*, 2019)), in order to evaluate the applicability of our findings from the *in vitro* system to the *in vivo* situation. In our new Fig. 3F-K and Fig. EV3G-H we show, that also *in vivo* on day E6.5 X-inactivation is highly heterogeneous and that specifically a cluster of *Fragilis*/*Ifitm3*-positive proximal epiblast cells, from which PGCs are specified, contains cell populations with (XaXi) and without (XaXa) X-inactivation (Fig. 3H, I). Furthermore, the cells with X-inactivation from the proximal epiblast cluster show attenuated gene silencing with many genes escaping X-inactivation, when compared to other epiblast clusters and extraembryonic cell types (Fig. 3J, K). This can be partially explained by the higher pluripotency factor expression when compared with posterior epiblast and extraembryonic cells (Fig. EV3H), which might contribute to the reduced degree of X-inactivation of PGC-competent proximal epiblast cells. This is in line with our observations from the *in vitro* system, where XGFP+ PGCLCs

display higher pluripotency gene expression than their XGFP- counterparts (Fig. 2E), ESC-like cell cycle profile (Fig. 2G) and a higher propensity to revert to pluripotent EGCs under 2i/LIF culture conditions (Fig. 2H, I). In sum, we conclude that the cell-to-cell heterogeneity and reduction in X-inactivation levels observed in our *in vitro* experiments reflects the situation of germ cell-competent proximal epiblast cells *in vivo* and is linked with a higher degree of pluripotent cell features.

Secondly, the X-GFP reporter system is not very sensitive to changes in the XCI levels. The authors manage to reach important conclusions despite these limitations, however fail to acknowledge them properly. The authors need to amend this and significantly change the flow of the paper. Moreover some important control experiments are also missing. After addressing these concerns I think this paper will be of great importance to the field of epigenetics and X inactivation.

AUTHOR'S RESPONSE: We agree with the concerns of the referee and have now highlighted the limitations of the reporter system by adding the following sentences:

Lines 168-173: "We do however note that EpiLCs still retained XGFP protein staining (Fig. 1E, F and EV1D, F), while XGFP transcripts were being downregulated (Fig. EV1C), indicating that protein stability of GFP gives a delayed read-out of X-inactivation kinetics. Nevertheless, at the PGCLC stage, the XGFP signal faithfully reflected the X-inactivation state as XGFP was only detected in cells without the X-inactivation-specific H3K27me3 spot (Fig. EV1D-F)."

Lines 445-447: "Taken together, in contrast to our observations based on the XGFP reporter, we observed by allele-specific RNA-Seq that reactivation of X-linked genes was almost complete by the time of meiotic entry."

Lines 492-495: "Although our XGFP marker has its limitations by showing a slight delay in response during kinetic changes such as downregulation during X-inactivation and upregulation during X-reactivation, it allowed us to isolate PGCLC populations with distinct developmental capacity."

In addition, we have now changed the flow of the paper significantly, by incorporating the *in vivo* analysis of X-inactivation in Fig. 3 and thereby showing the *in vivo* significance of our *in vitro* data. Furthermore, we swapped the order of Figures 5 and 6 (as suggested in point 1 below and also by reviewer #3 in point 8) by first discussing the X-reactivation kinetics in Fig. 5 and then by comparing the meiotic competence of XGFP+ and XGFP- PGCLCs functionally with new data in Fig. 6. Thereby we end the manuscript with the highlight of our study, which is that PGCLCs with proper X-inactivation/reactivation dynamics display significantly more efficient developmental progression towards meiosis.

Major points:

1) Results from Figure 4 H on its own are very confusing and counterintuitive. In fact they show that the X-GFP system is not suitable for measuring the levels of XCR. I suggest the following: rename the "GFP-" population to "GFP low" as on FACS there is still some signal. Immediately after this section describe the results now in Fig 6. It is important to show that in fact XCR takes place. This is also the moment when the major limitation of this system becomes apparent and should be appropriately acknowledged.

AUTHOR'S RESPONSE: We followed the excellent suggestion of the reviewer and renamed the XGFP- cells in Figures 4 and 5 to XGFPlow and also renamed the XGFP-reactivated cells to XGFPhigh in order to distinguish them from the constitutively active XGFP+ cells. Additionally, we changed the flow of this section by immediately following with the X-reactivation dynamics based on scRNA-Seq by making the previous Fig. 6 the new Fig. 5. In order to make it clearer that XCR takes place in these cells and to acknowledge that this is not fully reflected at this time point by the XGFP marker, we added the following sentence:

Lines 445-447: "Taken together, in contrast to our observations based on the XGFP reporter, we observed by allele-specific RNA-Seq that reactivation of X-linked genes was almost complete by the time of meiotic entry."

2) Replicates are missing throughout the manuscript. Most IF/FACS analysis were performed only once. This has to be amended and statistics included whenever possible.

AUTHOR'S RESPONSE: We have now added additional data, replicates and statistical analyses to many of the experiments including in Figure panels 1D, EV1E, EV1F, 6A-F. Furthermore, we added completely new Figure Panels EV1C, EV2C, EV2D, 3F-K, EV3F-H, EV4C, 6G, 6H.

3) Page 6. Conclusions from Fig EV1C are very convoluted and experiments are missing. Are there H3K27me3 foci in GFP+ve Sox2+ve cells? This staining needs to be performed. Can this be due to a high protein stability of GFP rather than the transcriptional status of the locus? This is an important limitation of this model and should be clearly addressed. It cannot be stated that H3K7me3 precedes gene silencing based on Zylicz et al 2019, as gene silencing at some loci is extremely rapid and quicker than H3K27me3 accumulation. Finally it is unclear what the first bar is in Fig ev1C.

AUTHOR'S RESPONSE: Following the reviewer's suggestion, we have now added the XGFP, SOX2, H3K27me3 triple staining to Fig. EV1D and performed the quantification Fig. EV1E-F. We came to the following conclusion:

Lines 168-173: "We do however note that EpiLCs still retained XGFP protein staining (Fig. 1E, F and EV1D, F), while XGFP transcripts were being downregulated (Fig. EV1C), indicating that protein stability of GFP gives a delayed read-out of X-inactivation kinetics. Nevertheless, at the

PGCLC stage, the XGFP signal faithfully reflected the X-inactivation state as XGFP was only detected in cells without the X-inactivation-specific H3K27me3 spot (Fig. EV1D-F)."

Furthermore, we have removed the statement about H3K27me3 and gene silencing based on the Zylicz paper. Also, we have added the EpiLC label to the first bar of the previous Fig. EV1C (now Fig. EV1E) which was missing originally.

4) The allelic ratio of 0.135 seems rather arbitrary. Are the conclusions holding when other thresholds are used? The degree of escape in Figure 3 is very high, how does that relate to findings from in vivo E6.5 epiblast? Published data should be used to check if this is not an in vitro artefact.

AUTHOR'S RESPONSE: We based our threshold of 0.135 on the first local minimum according to the distribution of genes in NPCs (see Fig. EV3B), a differentiated somatic cell type derived from the same parental ESC clone we used in this study, in which X-inactivation is almost complete (Bauer *et al*, 2021). Escapees of XCI are generally defined as genes being expressed from the Xi at greater than 10% of the levels of the active allele on the Xa (Peeters *et al*, 2014). Our cutoff for defining escapees of XCI is therefore very similar to previous studies in the field, which were often set at an allelic ratio of 0.15 (Janiszewski *et al*, 2019; Borensztein *et al*, 2017; Bauer *et al*, 2021; Xu *et al*, 2017). In order to see if this degree of escape / incomplete silencing relates to the situation *in vivo*, we analyzed E6.5 epiblast data by (Cheng *et al*, 2019). Thereby we found indeed also *in vivo* a high fraction of XaXa cells failing to undergo X-inactivation at all (Fig. 3I) and as well a high fraction of genes escaping X-inactivation / showing incomplete silencing in XaXi cells. Interestingly, this was specifically observed in Fragilis-positive proximal epiblast cells (Fig. 3F, G and EV3G), which are the cells in the epiblast with germ cell competence (Saitou *et al*, 2002; Lawson & Hage, 1994), while other parts of the epiblast showed a more complete degree of X-inactivation (Fig. 3H-K).

Finally, how does the early, late and escapee list of genes relate to published datasets?

AUTHOR'S RESPONSE: We now compared the early, late and escapee list of genes in our XGFP- PGCLCs with a published dataset of embryoid body (EB) differentiation (Marks *et al*, 2015), but did not observe a strong overlap (Fig. EV3F). This suggests that the inactivation kinetics of specific X-linked genes varies between different cell types and differentiation systems.

5) As it is, the discussion does not address key limitations of this system. It should be properly explained and discussed. Comparison to what happens and what could be different in vivo should also be discussed.

AUTHOR'S RESPONSE: In order to address the potential limitations of our system, we have now added the following statements in our discussion section:

Lines 492-495: "Although our XGFP marker has its limitations by showing a slight delay in response during kinetic changes such as downregulation during X-inactivation and upregulation during X-reactivation, it allowed us to isolate PGCLC populations with distinct developmental capacity."

Lines 522-525: "Nevertheless, we acknowledge that it would be important to validate our results during later stages of germ cell development *in vivo*. Particularly, whether PGCs that have failed to go through X-inactivation exist, and to what fate these cells would commit."

Minor points:

1) Line221-224: PC2 differences are never interpreted.

In response to the reviewer's request, we have now added Table EV4, which lists the PC1 and PC2 loadings of the PCA in Fig. 2B. When looking at the genes with contribution to the variance in PC1, which defined the developmental timing of *in vivo* and *in vitro* PGCs, we find among them meiosis genes such as *Dazl*, *Terb2* and *Hormad1*, but also genes related to pluripotency like *Dppa2* or processes such as cell migration, which could be explained by the migratory phenotype which PGCs display on there way to the gonads.
Although PC2 contains as well some genes with known germ cell functions such as *Nanos1*, *Eif2s3y* or *Meioc*, however, we did not observe a specific enrichment for germ cell genes. As the PC2 also did not contribute in this graph to the separation between our XGFP+ from XGFP- PGCLCs, we did not further comment on it in the manuscript.
Besides adding Table EV4, we also modified the text to the following:
Lines 196-198: "PCA revealed a trajectory where PC1 defined the developmental timing of *in vivo* and *in vitro* samples whereas PC2 did not greatly contribute to the separation of our *in vitro* PGCLCs. (PC loadings in Table EV4).

2) Figure 4 G: the dashed cells should be discussed in the text.

We are now showing the expression of the meiotic marker genes *Prdm9* and *Sycp3* in Fig. EV4C. As the cells in question do not express these key marker genes we added the following interpretation to the figure legend:
"(C) Marker gene expression of germ cell maturation clusters from rOvaries identified in this study, projected onto the in *vivo* UMAP plot. Cells on top left of the plot (in B and C), despite falling into the E16.5 cluster, represent deviant cells of abnormal character by not showing expression of late meiotic markers *Sycp3* and *Prdm9*."

3) From the text it remains unclear for me why the authors had to do the experiments in Fig 5. I'm guessing that they allow more robust meiotic activation but this should be properly explained in the text.

AUTHOR'S RESPONSE: The original Fig. 5 (now Fig. 6) aimed at showing and characterizing in more detail the capacity of XGFP- PGCLCs to further progress in meiosis in an *in vitro* expansion and meiotic differentiation system (Miyauchi *et al*, 2017). We have now added new data on XGFP+ cells in order to show the direct comparison of the meiotic progression potential of XGFP+ and XGFP- PGCLCs. Thereby we provide direct evidence that XGFP- PGCLCs have a significantly higher meiotic entry capacity than XGFP+ PGCLCs, confirming our hypothesis that progression through a controlled sequence of X-inactivation and subsequent X-reactivation is a predictor of high-quality PGCLCs with meiotic potential. Apart from the new data, we now better justified (in yellow), why we did this experiment (Lines 452-455):

"Our single-cell RNA-seq analysis showed an exclusive ability for XGFP- over XGFP+ PGCLCs to differentiate into mature germ cells with a meiotic transcriptional profile in the rOvary system. We therefore wanted to functionally compare their ability to enter meiosis and their capability to differentiate to more mature stages."

4) Figure 2D: the levels of Prdm14, Tfap2c, Blimp1, Esrrb are very similar between the GFP+ve and -ve cells. Such tiny differences should be validated using qPCR but with more replicates.

AUTHOR'S RESPONSE: We have followed the reviewer's request and added in panel Fig. EV2C the qPCR quantifications of the mentioned germ cell marker genes. All differences in gene expression mentioned between XGFP+ and XGFP- cells are statistically significant.

Referee #2:

Severino, Bauer and colleagues use an in vitro system of inducing germ cell fate and initial oogenesis from mouse embryonic stem cells to study the dynamics of X-inactivation/reactivation. Their hybrid cell line, harbouring biased X-inactivation and X-linked fluorescent reporters allows them to follow X-inactivation in this system with unprecedented detail.

I disagree with the authors that their experiments shows that X-inactivation dynamics impacts germ cell maturation and its potential to entry into meiosis (see below). I believe the major contribution of this manuscript is the high resolution, detailed view on the timing of X-inactivation & reactivation in the development of female germ cells. The work has its merits given the difficulties to perform these experiments in vivo, and the findings should be highlighted/discussed despite and in light of the shortcomings of the artificial in vitro system. I believe the work has significant appeal to the fields of X-inactivation and germline developmental biology, and I support the publication after minor experimental revision (see point 2) and major changes on the statements and discussion or additional experimental support for the claim that X-inactivation/reactivation may impact meiotic potential (see point 1).

AUTHOR'S RESPONSE: We thank the reviewer for highlighting the strengths as well as potential weaknesses of our study, which helped us in the revision process to improve our

manuscript substantially. We addressed the topics below in response to the specific points 1 + 2 both by adding new experimental data as well as by rewriting certain parts of our paper.

1. (First and second result sessions, lines 270 and 463-465) Claim that X-inactivation may be essential for proper PGCLC maturation and entry into meiosis:

Based on the presented experiments, the absence of XGFP+ in the pre-meiotic and meiotic populations obtained in vitro cannot be attributed to their failure in X-inactivation. The causal relationship between failure to inactivate X(mus) and improper maturation/meiosis progression has not been really tested. It is equally possible (and supported by their data on Figure 2) that XGFP+ cells represent an aberrant PGCLC population in which the failure to undergo X-inactivation is yet merely another manifestation of their aberrant state.

AUTHOR'S RESPONSE: We expanded our analysis by functionally comparing side by side the meiotic potential of XGFP- and XGFP+ PGCLCs in an *in vitro* model for meiotic induction (Miyauchi *et al*, 2017) using our *in vitro XRep* system, something which would be difficult to achieve *in vivo*. We thereby were able to confirm a significantly higher propensity of XGFP- PGCLCs to enter meiosis than XGFP+ cells (new data in Fig. 6C), which mostly showed an abnormal proliferative phenotype instead (Fig. 6G, H). Nevertheless, we agree with the reviewer that this is not necessarily formal proof of causality for X-inactivation per se being the sole driver of the phenotypic differences. Alternatively, the X-inactivation status could also be a sensitive biomarker for normal versus abnormal PGCLCs. We therefore tried to be careful in our choice of words throughout our manuscript to make sure not to state that X-inactivation may be the definitive source of PGCLCs' meiotic potential. Indeed, we already stated in our discussion previously:

Lines 526-528:"It remains an open question, what could be the potential role of X-inactivation for proper PGCLC development and if it is a driver or, alternatively, a diagnostic mark for meiotic competence of germ cells. "

and:

Lines 562-565: "To which degree this may be a cause or consequence of the X-inactivation status in PGCLCs and how X-linked gene dosage might affect female germ cell development will need to be addressed by future studies, for example by testing the meiotic potential of *Xist* knockout cells."

In order to make this now even clearer, we made additional changes to the text:

Previously lines 103-105: "This suggests that both dosage control and epigenetic reprogramming of the X chromosome may be critical steps required for female germ cell's developmental progression towards meiosis and oogenesis."

We changed this into (new lines 114-116): "This suggests that both dosage control and epigenetic reprogramming of the X chromosome may be critical indicators for female germ cell's developmental potential to progress towards meiosis and oogenesis."

Previously line 270: "... indicating that X-inactivation could be necessary for correct PGCLC maturation."

We changed this into (new lines 249-250): "This indicates that X-inactivation could be necessary for correct PGCLC maturation, or, alternatively, be a sensitive biomarker of high-quality PGCLCs."

Previously lines 463-465: "However, our data suggest that X-inactivation is important for proper germ cell maturation and entry into meiosis, as germ cells originating from constitutively active XGFP+ PGCLCs failed to acquire a meiotic transcriptional profile."

We changed this into (new lines 417-420): "However, our data suggest that X-inactivation may be either functionally important, or alternatively, a predictive indicator for subsequent germ cell maturation and entry into meiosis, as germ cells originating from constitutively active XGFP+ PGCLCs failed to acquire a meiotic transcriptional profile."

A percentage of aberrant PGCLCs may be an unsurmountable shortcoming of the in vitro PGCLC system, or it may be that aberrant reprogramming also occurs in vivo. Perhaps the massive attrition of foetal oocytes, and the somewhat less extensive attrition of gonocytes observed in vivo reflects this phenomenon. I think the definite experiment would involve testing if the implantation of blastocysts made chimeric with Xist KO ES cells could lead to females in which the ES cells contributes to oogonia at the meiotic prophase I. However, this seems like an unreasonable request for the scope of this manuscript.

AUTHOR'S RESPONSE: We agree with the referee that the *in vivo* chimeric *Xist* KO experiment would be an elegant gold standard way to test our hypothesis. However, we also concede that this type of experiment would be out of the scope of this manuscript as it would take a very long time to generate the necessary materials, set up the experimental system and analyze the meiotic germ cell phenotype. Nevertheless, it would be an interesting follow-up project of our study and we thank the referee for the excellent suggestion.

Perhaps a more feasible experiment would be to produce a Xist KO (or X-inactivation deficient) ES cell line on top of their XRep, follow with the EpiLC-PGCLC differentiation, and use the entirety of SSEA1/CD61 cells-positive cells in the rOvary reconstitution. Since in Xist WT XRep cells the XGFP- (meiotic competent) reliably constitute 60% of their PGCLC population, the number of cells that successfully enter meiosis should roughly follow the 60/40% of XGFP-/XGFP+. Despite the challenges of making such an experiment quantitative to an extent where these proportions can be followed throughout the in vitro differentiation protocols, it would at least provide some evidence that X-inactivation is required for PGCLC development/maturation.

AUTHOR'S RESPONSE: We agree that producing an *Xist KO* in our *XRep* cell line would allow us to directly address the question of causality for X-inactivation in meiotic potential. In fact, we are in the process of engineering and testing such a cell line for the follow-up project of this paper. On the other hand, we anticipate major challenges for the successful differentiation of this cell line into PGCLCs, as extensive X-chromosome loss (Colognori *et al*, 2020) and defects in pluripotency exit (Schulz *et al*, 2014) are known phenomena to occur during differentiation of *Xist* KO ES cell lines. This might therefore make the analysis of the meiotic phenotype non-trivial and will take a considerable amount of time and effort, outside a realistic time frame of the revision period of this paper.

In any case, I think it would be fair to discuss these potential shortcomings more openly. Instead, the authors attempt to sell the system as more advantageous, and that their data supports a model where X-chromosome dosage compensation kinetics and germline development are tied under a functional link (lines 613-628).

AUTHOR'S RESPONSE: We concur with the point of the reviewer that it is important to be more open about potential shortcomings. While we favor a direct role of X-dosage compensation in ensuring PGCLC quality we need to stress that there is also the alternative hypothesis that abnormal PGCLC differentiation can result in a lack of X-inactivation instead. In addition to the clarifying sentences in the responses mentioned above, we therefore modified the discussion section:

After the passage mentioned by the reviewer (previously lines 626-628, now lines 520-522): "Thus our data supports the idea of a potential functional link between appropriate X-chromosome dosage compensation kinetics and developmental progression during mammalian germ cell maturation."

We added the sentence (lines 522-525): "Nevertheless, we acknowledge that it would be important to validate our results during later stages of germ cell development *in vivo*. Particularly, whether PGCs that have failed to go through X-inactivation exist, and to what fate these cells would commit."

Furthermore, in lines 562-565, we added: "To which degree this may be a cause or consequence of the X-inactivation status in PGCLCs and how X-linked gene dosage might affect female germ cell development will need to be functionally addressed by future studies, for example by testing the meiotic potential of *Xist* knockout cells."

2. Related to the shortcomings of the in vitro PGCLC system for studying X-inactivation: what is the percentage of XGFP- and XGFP+ cells at the day 4 of EpiLCs? Based on Figure 1F it seems that X-inactivation has barely started at the point where cell fate is pushed towards PGCLCs (although it seems the figure shows only the percentage of gated PGC-marker positive, SSEA1/CD61 cells, which should not be present anyway at this point). It would be

important to show this data for the entirety of EpiLCs in comparison and discuss how does this compare to the dynamics of X-inactivation in vivo, at the stage when PGCs are specified.

AUTHOR'S RESPONSE: There must have been a misunderstanding, as in fact we have quantified the percentage of XGFP- and XGFP+ cells by FACS in ESCs, EpiLCs and d1 full bodies for all cells and only in the PGCLC timepoints d2 to d5 for the SSEA1+/CD61+ population. To make this more evident, we have now relabeled the panel in Fig. 1F and clearly marked where only the SSEA1+/CD61+ cells have been taken into account and rewritten the figure legend:
"XGFP percentages in ESCs, EpiLCs and d1 full bodies are calculated from the entire cell population, while in PGCLC d2 to PGCLC d5 are calculated from SSEA1+/CD61+ PGCLCs as indicated."

Furthermore, while it is true that almost all EpiLCs are XGFP+ (Fig. 1F), the majority of cells show a H3K27me3 spot (Fig. EV1D-F) and as well show a moderate downregulation of XGFP RNA (Fig. EV1C) and of X-linked genes (Fig. 3A-C), indicating initiation of X-inactivation. Therefore the high percentage (97.7%) of XGFP+ EpiLCs is due to the prolonged half-life of GFP protein, which does not accurately reflect the downregulation kinetics at the RNA level. We clarified this in the text by adding the following sentences:

Results, lines 168-171: "We do however note that EpiLCs still retained XGFP protein staining (Fig. 1E, F and EV1D, F), while XGFP transcripts were being downregulated (Fig. EV1C), indicating that protein stability of GFP gives a delayed read-out of X-inactivation kinetics."
Discussion, lines 492-495: " Although our XGFP marker has its limitations by showing a slight delay in response during kinetic changes such as downregulation during X-inactivation and upregulation during X-reactivation, it allowed us to isolate PGCLC populations with distinct developmental capacity."

Finally, we acknowledge that our first manuscript version was based on data generated using our *in vitro* XRep system and that a validation of our findings *in vivo* would greatly enhance the significance of our results. For that reason, we have now reanalyzed *in vivo* allelic single-cell RNA-Seq data from E6.5 mouse embryos (Cheng *et al*, 2019) and added this as new panels Fig. 3F-K and Fig. EV3G-H. We could thereby confirm that also *in vivo*, cells with germ cell competence in the proximal epiblast around the time of PGC specification show heterogeneity with respect to X-inactivation and a lower degree of gene silencing when compared to other cells in the epiblast or in extraembryonic lineages. Thus our findings from the *in vitro* system are recapitulated *in vivo* as well.

Referee #3:

The manuscript characterizes the X inactivation and reactivation dynamics during in vitro differentiation of female germ cells. To this end, a female mouse embryonic stem cell line (XRep) is used that allows allele-specific monitoring of X inactivation by allele-specific RNA-seq and by flow cytometry through X-linked fluorescent reporters. The manuscript reports the dynamics of X inactivation and X reactivation during differentiation of the XRep cell line towards female germ cells, assessed by bulk and single-cell RNA-seq. To understand how the X inactivation status of a cell correlates with its differentiation potential, the X-linked fluorescent reporters are used to sort cells according to their X inactivation status after PGCLC differentiation. Cells that have not yet undergone X inactivation are found to be less differentiated and to not be able to progress towards meiosis. While this is an interesting observation, it is not clear how it should be interpreted. As also pointed out in the discussion section, it is unclear whether there is a functional link between X inactivation status and differentiation potential or whether the two processes are just temporally correlated. Moreover, I am not sure how the specific parameters of the in vitro differentiation systems (e.g. length of culture in a specific condition) affect the result and to what extent they are thus indicative of the extendedprocess in vivo.

AUTHOR'S RESPONSE: We thank the reviewer for the assessment of our manuscript. We addressed the specific points raised below.

Major points

1. When assessing GFP expression during the differentiation procedure, it looks like EpiLCs at day 4 are just initiating X inactivation (Fig. 1F, 3B). Since these cells in vivo have already undergone X inactivation, the in vitro system used does not seem to recapitulate this correctly. Maybe the EpiLC culture should be extended or the differentiation protocol should be optimized?

AUTHOR'S RESPONSE: We have now revisited the *in vivo* X-inactivation status in mouse E6.5 epiblast by analyzing published single-cell RNA Seq data (Cheng *et al*, 2019). Thereby we found (new panels Fig 3F-K) that in fact also *in vivo* around the time point of PGC specification (Ohinata *et al*, 2005), specifically germ-cell competent proximal epiblast cells show heterogeneous and incomplete X-inactivation in contrast to other cells of the epiblast or in extraembryonic tissues, which do not contribute to the germ cell lineage. Thereby we provide evidence that the incomplete X-inactivation state in our EpiLCs is not merely an *in vitro* phenomenon, but rather a reflection of germ cell competence within the *in vivo* epiblast.
Furthermore, when setting up the conditions of our *in vitro* PGCLC differentiation system, we noticed that suboptimal timing of EpiLC differentiation would be prohibitive for efficient PGCLC specification. This is in line with published reports that extended EpiLC differentiation into EpiSCs would interfere with PGCLC specification (Hayashi *et al*, 2011) due to a loss of germline competency by epigenetic decommissioning of germ cell gene enhancers during extended EpiLC differentiation (Bleckwehl *et al*, 2021). For that reason and the fact that our EpiLCs

represent a similar X-inactivation state as the germ cell competent epiblast *in vivo*, we decided not to deviate from our EpiLC differentiation timing prior to PGCLC induction.

2. When the XGFP+ cells are sorted at day 5 of PGCLC differentiation, these represent 7% of cells that appear to be slightly delayed in differentiation compared to the XGFP- cells that have undergone X inactivation (Fig. 2, EV2). In the initial characterization of the system in Fig. 1F, 40% of cells are XGFP+ at day 5, suggesting less efficient differentiation in that experiment. Would then a longer differentiation towards PGCLC allow full X inactivation and thus more efficient differentiation towards the germ line?

AUTHOR'S RESPONSE: We agree with the reviewer that a longer differentiation time of PGCLCs might result in a further progression in X-inactivation. For exactly that reason we optimized the originally published PGCLC differentiation protocols developed in the Saitou lab (Hayashi *et al*, 2011, 2012; Nakaki *et al*, 2013) for our X-inactivation study and already extended both the EpiLC differentiation from the usual 2 days to 4 days as well as the PGCLC differentiation from normally 3-4 days to 5 days (as justified in results lines 148-152). Longer transcription-factor induced PGCLC differentiation for 6 days has been shown to lead to abnormal cells unsuitable for downstream gametogenesis (Nakaki *et al*, 2013). For that reason and in order not to deviate excessively from the timing and gene expression profile of PGCs *in vivo*, which our cells faithfully recapitulated (Fig. 2B), we refrained from further extending our differentiation timing.

3. The manuscript suggests that the xGFP+ cells, which later exhibited impaired differentiation, might "correspond to an aberrant PGCLC state with similarities to ESCs" (p. 10, line 250). I don't understand how this conclusion is reached, since the presented data rather suggests that the differentiation speed is heterogeneous and that these cells are somewhat delayed in their differentiation, and might actually be able to further differentiate, if cultured under PGCLC conditions for longer?

AUTHOR'S RESPONSE: As mentioned in the response to point 2 above it would be unfeasible to alter the PGCLC differentiation timing without disrupting their normal *in vivo*-like development. Furthermore, it becomes clear from our overall data, that XGFP+ PGCLCs on one hand display properties related to pluripotency (cell cycle profile, elevated pluripotency gene expression and higher propensity to revert to EGCs in 2i+LIF conditions than XGFP- PGCLCs). On the other hand however, XGFP+ PGCLCs cluster closer to XGFP- PGCLCs than to ESCs in a PCA analysis (Fig. 2A), show higher expression of late germ cell marker genes than XGFP- PGCLCs (see our new data panel EV2D) and also place later than XGFP- PGCLCs on a developmental trajectory based on a PCA with *in vivo* PGCs (Fig. 2B). Therefore XGFP+ PGCLCs are not simply less differentiated ES cell-like cells, but rather represent a more complex abnormal phenotype with both pluripotency-related as well as late PGCLC-like features. In order to make this point clearer, we changed the following sentences:

Previously line 250: "As our transcriptomics and cell cycle analysis suggested that XGFP+ PGCLCs could correspond to an aberrant PGCLC state with similarities to ESCs… "

into now lines 228-229: "As our transcriptomics and cell cycle analysis suggested that XGFP+ PGCLCs could correspond to an aberrant PGCLC state with properties related to ESCs… "

And previously lines 268-270: "this suggests that XGFP+ PGCLCs may correspond to an aberrant PGCLC state of pluripotent stem cell-like character.

Into now lines 247-249:"this suggests that XGFP+ PGCLCs may correspond to an aberrant PGCLC state with pluripotent stem cell-related features."

4. In Fig. 5 cells are differentiated beyond the PGCLC state, which leads to X reactivation in XGFP- cells. It would be useful to see this test for XGFP+ cells as well to support the idea that X inactivation is required for germ cell specification.

AUTHOR'S RESPONSE: We thank the reviewer for the excellent suggestion and we now performed side-by-side comparison experiments of XGFP- and XGFP+ PGCLCs to test if cells with proper X-inactivation kinetics have a higher meiotic competence when subjected to an *in vitro* meiotic induction protocol (Miyauchi *et al*, 2017). In these new data presented in Fig. 6, we could indeed observe that XGFP- PGCLCs were significantly more likely to enter meiosis than XGFP+ PGCLCs (Fig. 6C). In contrast, XGFP+ PGCLCs frequently displayed an abnormal proliferation phenotype (Fig. 6G-H).

Minor points

5. I find the color scale used for the heatmaps misleading (Fig. 3C, 6A, 6C) as it is centered around 0.13. I would suggest to use a two-color system centered around 0.5, as this corresponds of biallelic expression.

AUTHOR'S RESPONSE: We have carefully considered and tested different heatmap color schemes as suggested by the reviewer. However, we noticed that when centered around 0.5, the heatmaps became a lot less informative, as they would show only a single color as almost all our data (except for *Xist*, which is expressed from Xmus and occasional outlier genes) are below 0.5 due to the skewed inactivation of the Xmus chromosome in our *Tsix-Stop XRep* cell line. As explained above in response to major point 4 of reviewer 1, we picked 0.135 as X-inactivation cutoff based on the distribution of inactivated genes in NPCs and according to the accepted standard in the field that allelic ratios below around 0.15 represent inactive and above active genes (Janiszewski *et al*, 2019; Borensztein *et al*, 2017; Bauer *et al*, 2021; Xu *et al*, 2017; Peeters *et al*, 2014). The main purpose of our chosen color palette was to clearly discriminate visually between active (green) and inactive genes (red), which allows us to categorize into early, late inactivating/reactivating or escapee genes at any given time point in our analysis (Fig. 3C, 3J, 5A, 5C). Picking a different threshold for color change than the X-inactivation cutoff of 0.135 would be a more arbitrary choice and would not convey this important information when genes are considered ON or OFF.

6. Although the fluorescent reporters are a useful tool to assess X inactivation, their (binary) activity cannot be used to discern whether a cell has undergone X inactivation or not. Since X inactivation as well as X reactivation occurs with variable dynamics across genes, it can be misleading to describe it as a binary process. On p. 6, line 153 for example it is stated that "XGFP+ PGCLCs failed to undergo X-linked gene silencing...". In such instances it would be more precise to state that cells failed to undergo silencing of the X-linked GFP transgene.

AUTHOR'S RESPONSE: We absolutely agree with the reviewer, in particular as we found differences in categorizing cells as active or inactive when comparing the XGFP fluorescence status with X-linked gene expression based on RNA-levels during periods of X-linked gene silencing or reactivation (Fig. 1F vs. EV1C and 3B+C; Fig. 4C vs. 5A). This can be explained both by the reviewer's suggestion that individual X-linked genes show different inactivation/reactivation kinetics when compared to XGFP and as well by the delay in reaction time of the XGFP marker due to the extended GFP protein half-life during X-inactivation and lag in fluorescence acquisition during X-reactivation. We therefore made the following clarifying additions to the text:

Lines 168-173: "We do however note that EpiLCs still retained XGFP protein staining (Fig. 1E, F and EV1D, F), while XGFP transcripts were being downregulated (Fig. EV1C), indicating that protein stability of GFP gives a delayed read-out of X-inactivation kinetics. Nevertheless, at the PGCLC stage, the XGFP signal faithfully reflected the X-inactivation state as XGFP was only detected in cells without the X-inactivation-specific H3K27me3 spot (Fig. EV1D-F)."

Lines 445-447: "Taken together, in contrast to our observations based on the XGFP reporter, we observed by allele-specific RNA-Seq that reactivation of X-linked genes was almost complete by the time of meiotic entry."

Lines 492-495: "Although our XGFP marker has its limitations by showing a slight delay in response during kinetic changes such as downregulation during X-inactivation and upregulation during X-reactivation, it allowed us to isolate PGCLC populations with distinct developmental capacity."

7. According to the legend Fig. 1F shows all live cells, but the XGFP percentages is only calculated within SSEA1+/CD61+ cells. Is this correct? I would suggest to calculate the percentages for the cells shown in the plot.

AUTHOR'S RESPONSE: We appreciate that this point was not made very clear in our manuscript, as it was also mentioned by reviewer 2 in point 2. In fact, we have quantified the percentage of XGFP- and XGFP+ cells by FACS in ESCs, EpiLCs and d1 full bodies for all cells and only in the PGCLC timepoints d2 to d5 for the SSEA1+/CD61+ population. To make this more evident, we have now relabeled the panel in Fig. 1F and clearly marked where only the SSEA1+/CD61+ cells have been taken into account and rewritten the figure legend:

"XGFP percentages in ESCs, EpiLCs and d1 full bodies are calculated from the entire cell population, while in PGCLC d2 to PGCLC d5 are calculated from SSEA1+/CD61+ PGCLCs as indicated."

Other points

8. I would suggest to invert the order of Fig. 5 and Fig. 6, since the characterization of XCI by allele-specific scRNA-seq analysis in Fig. 6 follows logically from Fig. 4H, which looks at the X inactivation status in the same data set by flow cytometry.

AUTHOR'S RESPONSE: We thank the reviewer for the excellent suggestion and we have followed the advice and swapped the order of Fig. 5 and Fig. 6. This now greatly improved the flow of our manuscript.

9. The font size of the labels in some of the heatmaps are too small.

AUTHOR'S RESPONSE: We have now increased the font sizes in the heatmaps in Figures 2E, 3C, 3J, 5C and in the GO term analysis in Fig. 2F. These are now the maximum font sizes possible due to the number of genes listed in those plots. As they are vector graphics, they are clearly readable when zooming into the plots.

REFERENCES

Bauer M, Vidal E, Zorita E, Üresin N, Pinter SF, Filion GJ & Payer B (2021) Chromosome compartments on the inactive X guide TAD formation independently of transcription during X-reactivation. *Nat Commun* 12: 2020.07.02.177790

Bleckwehl T, Crispatzu G, Schaaf K, Respuela P, Bartusel M, Benson L, Clark SJ, Dorighi KM, Barral A, Laugsch M, *et al* (2021) Enhancer-associated H3K4 methylation safeguards in vitro germline competence. *Nat Commun* 12: 1–19

Borensztein M, Okamoto I, Syx L, Guilbaud G, Picard C, Ancelin K, Galupa R, Diabangouaya P, Servant N, Barillot E, *et al* (2017) Contribution of epigenetic landscapes and transcription factors to X-chromosome reactivation in the inner cell mass. *Nat Commun* 8: 1297

Cheng S, Pei Y, He L, Peng G, Reinius B, Tam PP, Jing N & Deng Q (2019) Single-Cell RNA-Seq Reveals Cellular Heterogeneity of Pluripotency Transition and X Chromosome Dynamics during Early Mouse Development. *Cell Rep* 26: 2593–2607.e3

Colognori D, Sunwoo H, Wang D, Wang C-Y & Lee JT (2020) Xist Repeats A and B Account for Two Distinct Phases of X Inactivation Establishment. *Dev Cell* 54: 21–32.e5

Hayashi K, Ogushi S, Kurimoto K, Shimamoto S, Ohta H & Saitou M (2012) Offspring from oocytes derived from in vitro primordial germ cell-like cells in mice. *Science* 338: 971–975

Hayashi K, Ohta H, Kurimoto K, Aramaki S & Saitou M (2011) Reconstitution of the mouse germ cell specification pathway in culture by pluripotent stem cells. *Cell* 146: 519–532

Janiszewski A, Talon I, Chappell J, Collombet S, Song J, De Geest N, To SK, Bervoets G, Marin-Bejar O, Provenzano C, *et al* (2019) Dynamic reversal of random X-Chromosome inactivation during iPSC reprogramming. *Genome Res*

Lawson KA & Hage WJ (1994) Clonal analysis of the origin of primordial germ cells in the mouse. *Ciba Found Symp* 182: 68–84; discussion 84–91

Marks H, Kerstens HHD, Barakat TS, Splinter E, Dirks RAM, van Mierlo G, Joshi O, Wang S-Y, Babak T, Albers CA, *et al* (2015) Dynamics of gene silencing during X inactivation using allele-specific RNA-seq. *Genome Biol* 16: 149

Miyauchi H, Ohta H, Nagaoka S, Nakaki F, Sasaki K, Hayashi K, Yabuta Y, Nakamura T, Yamamoto T & Saitou M (2017) Bone morphogenetic protein and retinoic acid synergistically specify female germ-cell fate in mice. *EMBO J* 36: 3100–3119

Nakaki F, Hayashi K, Ohta H, Kurimoto K, Yabuta Y & Saitou M (2013) Induction of mouse germ-cell fate by transcription factors in vitro. *Nature*

Ohinata Y, Payer B, O'Carroll D, Ancelin K, Ono Y, Sano M, Barton SC, Obukhanych T, Nussenzweig M, Tarakhovsky A, *et al* (2005) Blimp1 is a critical determinant of the germ cell lineage in mice. *Nature* 436: 207–213

Peeters SB, Cotton AM & Brown CJ (2014) Variable escape from X-chromosome inactivation: identifying factors that tip the scales towards expression. *Bioessays* 36: 746–756

Saitou M, Barton SC & Surani MA (2002) A molecular programme for the specification of germ cell fate in mice. *Nature* 418: 293–300

Schulz EG, Meisig J, Nakamura T, Okamoto I, Sieber A, Picard C, Borensztein M, Saitou M, Blüthgen N & Heard E (2014) The two active X chromosomes in female ESCs block exit from the pluripotent state by modulating the ESC signaling network. *Cell Stem Cell* 14: 203–216

Xu J, Carter AC, Gendrel A-V, Attia M, Loftus J, Greenleaf WJ, Tibshirani R, Heard E & Chang HY (2017) Landscape of monoallelic DNA accessibility in mouse embryonic stem cells and neural progenitor cells. *Nat Genet*: 1–13

1st Revision - Editorial Decision 27th Mar 2022

Dear Dr Payer,

Thank you for submitting your revised manuscript (EMBOJ-2021-109457R) to The EMBO Journal. Your amended study was sent back to the three referees for re-evaluation, and we have received comments from all of them, which I enclose below. As you will see, the referees stated that the issues raised have been adequately addressed and that they are broadly now in favour of publication, pending minor revision.

Thus, we are pleased to inform you that your manuscript has been accepted in principle for publication in The EMBO Journal.

Please consider the remaining points of referee #1 carefully, and address these by adjusting data display and introducing textual changes where appropriate.

In addition, we need you to take care of a number of issues related to formatting and data representation as detailed below, which should be addressed at re-submission.

Please contact me at any time if you have additional questions related to below points.

Thank you for giving us the chance to consider your manuscript for The EMBO Journal. I look forward to your final revision.

Again, please contact me at any time if you need any help or have further questions.

Best regards,

Daniel Klimmeck

Daniel Klimmeck PhD
Senior Editor
The EMBO Journal

Formatting changes required for the revised version of the manuscript:

>> Adjust the title of the 'Competing Interests' section to 'Disclosure and Competing Interests Statement'.

>> Please make sure the funding information stated in the manuscript and online system are consistent and complete.

>> Dataset EV legends: Dataset EV1 & EV2 are fine. Tables EV3 & EV4 should also be named Dataset files as they are several sheets long. The legends should be removed from the manuscript file.

>> Please introduce a GEO database hyperlink in the data accessibility section, and remove the referee token, also release privacy from the data set.

>> Add a separate 'Statistical analysis' section to your manuscript, detailing the algorithms applied.

>> Recheck publication status of the bioRxiv references for Lentini et al. 2021 and Naik et al. 2021 for updates.

>> Add mouse strain and animal welfare information to the Author Checklist.

>> Please consider additional changes and comments from our production team as indicated by the .doc file enclosed and leave changes in track mode.

Please remember: Digital image enhancement is acceptable practice, as long as it accurately represents the original data and conforms to community standards. If a figure has been subjected to significant electronic manipulation, this must be noted in the figure legend or in the 'Materials and Methods' section. The editors reserve the right to request original versions of figures and the original images that were used to assemble the figure.

Further information is available in our Guide For Authors: https://www.embopress.org/page/journal/14602075/authorguide

We realize that it is difficult to revise to a specific deadline. In the interest of protecting the conceptual advance provided by the work, we recommend a revision within 3 months (25th Jun 2022). Please discuss the revision progress ahead of this time with the editor if you require more time to complete the revisions. Use the link below to submit your revision:

Link Not Available

Referee #1:

The revised manuscript by Severino et al. now contains significant additional results and analysis. The text also implemented more nuanced data interpretations. The authors have addressed most of my comments. Importantly, by reanalyzing published data they have revealed an interesting heterogeneity of XCI status in vivo within the PGC-competent epiblast cells. I support the publication of this manuscript in EMBO but I suggest the implementation of two minor changes:

-Figure 6B: each cell in the IF has red staining. It would be useful for the reader to know which pattern of staining you scored and maybe include arrows to show such cells.
-Page 9 line 297-302 this text is largely a repetition from page 8.

Referee #2:

Severino and colleagues have addressed most of the concerns raised by myself and other reviewers. Re-analysis of published in vivo datasets and a test of meiotic induction XGFP- and XGFP+ PGCLCs substantially reinforced some of the weaker aspects of the first manuscript. The manuscript has also been substantially reworked to reflect the shortcomings of the in vitro system, and to account for alternative hypothesis related to the causality between X-inactivation state and PGC maturation/meiotic potential. Short of conducting riskier and lengthier experiments, this is the best the authors could provide in face of the criticism presented. I believe the manuscript is now more balanced and provide a useful view on the importance and dynamics of X-inactivation in primordial germ cell development.

Referee #3:

The authors addressed all my concerns in the revised version of the manuscript. I support its publication in the present form.

The authors performed the requested editorial changes.

Dear Dr Payer,

Thank you for submitting the revised version of your manuscript. I have now evaluated your amended manuscript and concluded that the remaining minor concerns have been sufficiently addressed.

Thus, I am pleased to inform you that your manuscript has been accepted for publication in the EMBO Journal.

Please note that it is EMBO Journal policy for the transcript of the editorial process (containing referee reports and your response letter) to be published as an online supplement to each paper.

Also, in case you might NOT want the transparent process file published at all, you will also need to inform us via email immediately. More information is available here:
https://www.embopress.org/page/journal/14602075/authorguide#transparentprocess

----

Please note that in order to be able to start the production process, our publisher will need and contact you regarding the following forms:

- PAGE CHARGE AUTHORISATION (For Articles and Resources)
http://onlinelibrary.wiley.com/journal/10.1002/(ISSN)1460-2075/homepage/tej_apc.pdf

- LICENCE TO PUBLISH (for non-Open Access)

Your article cannot be published until the publisher has received the appropriate signed license agreement. Once your article has been received by Wiley for production you will receive an email from Wiley's Author Services system, which will ask you to log in and will present them with the appropriate license for completion.

- LICENCE TO PUBLISH for OPEN ACCESS papers

Authors of accepted peer-reviewed original research articles may choose to pay a fee in order for their published article to be made freely accessible to all online immediately upon publication. The EMBO Open fee is fixed at $5,500 (+ VAT where applicable).

We offer two licenses for Open Access papers, CC-BY and CC-BY-NC-ND.
For more information on these licenses, please visit:
http://creativecommons.org/licenses/by-nc-nd/3.0/deed.en_US

- PAYMENT FOR OPEN ACCESS papers

You also need to complete our payment system for Open Access articles. Please follow this link and select EMBO Journal from the drop down list and then complete the payment process: https://authorservices.wiley.com/bauthor/onlineopen_order.asp

Notably, please be reminded that under the DEAL agreement of European scientific institutions with our publisher Wiley, you could be eligible for free publication of your article in the open access format. Please contact either the administration at your institution or Wiley (embojournal@wiley.com) to clarify further questions.

Should you be planning a Press Release on your article, please get in contact with embojournal@wiley.com as early as possible, in order to coordinate publication and release dates.

On a different note, I would like to alert you that EMBO Press is currently developing a new format for a video-synopsis of work published with us, which essentially is a short, author-generated film explaining the core findings in hand drawings, and, as we believe, can be very useful to increase visibility of the work. This has proven to offer a nice opportunity for exposure i.p. for the first author(s) of the study. Please see the following link for representative examples and their integration into the article web page:
https://www.embopress.org/video_synopses
https://www.embopress.org/doi/full/10.15252/embj.2019103932

Please let me know, should you be interested to engage in commissioning a similar video synopsis for your work. According operation instructions are available and intuitive.

Finally, we have noted that the submitted version of your article is also posted on the preprint platform bioRxiv. We would appreciate if you could alert bioRxiv on the acceptance of this manuscript at The EMBO Journal in order to allow for an update of the entry status. Thank you in advance!

If you have any questions, please do not hesitate to call or email the Editorial Office.

Thank you for this contribution to The EMBO Journal and congratulations on a successful publication! Please consider us again in the future for your most exciting work.

Kind regards,

Daniel Klimmeck

Daniel Klimmeck, PhD
Senior Editor
The EMBO Journal
EMBO
Postfach 1022-40
Meyerhofstrasse 1
D-69117 Heidelberg
contact@embojournal.org
Submit at: http://emboj.msubmit.net

EMBO Press Author Checklist

Corresponding Author Name: Bernhard Payer	
Journal Submitted to: EMBO Journal	
Manuscript Number: EMBOJ-2021-109457R	

USEFUL LINKS FOR COMPLETING THIS FORM
The EMBO Journal - Author Guidelines
EMBO Reports - Author Guidelines
Molecular Systems Biology - Author Guidelines
EMBO Molecular Medicine - Author Guidelines

Reporting Checklist for Life Science Articles (updated January 2022)
This checklist is adapted from Materials Design Analysis Reporting (MDAR) Checklist for Authors. MDAR establishes a minimum set of requirements in transparent reporting in the life sciences (see Statement of Task: 10.31222/osf.io/9sm4x). Please follow the journal's guidelines in preparing your manuscript.
Please note that a copy of this checklist will be published alongside your article.

Abridged guidelines for figures
1. Data
The data shown in figures should satisfy the following conditions:
- the data were obtained and processed according to the field's best practice and are presented to reflect the results of the experiments in an accurate and unbiased manner.
- ideally, figure panels should include only measurements that are directly comparable to each other and obtained with the same assay.
- plots include clearly labeled error bars for independent experiments and sample sizes. Unless justified, error bars should not be shown for technical replicates.
- if n<5, the individual data points from each experiment should be plotted. Any statistical test employed should be justified.
- Source Data should be included to report the data underlying figures according to the guidelines set out in the authorship guidelines on Data Presentation.

2. Captions
Each figure caption should contain the following information, for each panel where they are relevant:
- a specification of the experimental system investigated (eg cell line, species name).
- the assay(s) and method(s) used to carry out the reported observations and measurements.
- an explicit mention of the biological and chemical entity(ies) that are being measured.
- an explicit mention of the biological and chemical entity(ies) that are altered/varied/perturbed in a controlled manner.
- the exact sample size (n) for each experimental group/condition, given as a number, not a range;
- a description of the sample collection allowing the reader to understand whether the samples represent technical or biological replicates (including how many animals, litters, cultures, etc.).
- a statement of how many times the experiment shown was independently replicated in the laboratory.
- definitions of statistical methods and measures:
  - common tests, such as t-test (please specify whether paired vs. unpaired), simple χ2 tests, Wilcoxon and Mann-Whitney tests, can be unambiguously identified by name only, but more complex techniques should be described in the methods section;
  - are tests one-sided or two-sided?
  - are there adjustments for multiple comparisons?
  - exact statistical test results, e.g., P values = x but not P values < x;
  - definition of 'center values' as median or average;
  - definition of error bars as s.d. or s.e.m.

Please complete ALL of the questions below.
Select "Not Applicable" only when the requested information is not relevant for your study.

Materials

Newly Created Materials	Information included in the manuscript?	In which section is the information available? (Reagents and Tools Table, Materials and Methods, Figures, Data Availability Section)
New materials and reagents need to be available; do any restrictions apply?	Not Applicable	No restrictions apply.

Antibodies	Information included in the manuscript?	In which section is the information available? (Reagents and Tools Table, Materials and Methods, Figures, Data Availability Section)
For **antibodies** provide the following information:<br>- Commercial antibodies: RRID (if possible) or supplier name, catalogue number and or/clone number<br>- Non-commercial: RRID or citation	Yes	Materials and Methods

DNA and RNA sequences	Information included in the manuscript?	In which section is the information available? (Reagents and Tools Table, Materials and Methods, Figures, Data Availability Section)
**Short novel DNA or RNA including primers, probes**: provide the sequences.	Yes	Materials and Methods. Table 1.

Cell materials	Information included in the manuscript?	In which section is the information available? (Reagents and Tools Table, Materials and Methods, Figures, Data Availability Section)
**Cell lines:** Provide species information, strain. Provide accession number in repository **OR** supplier name, catalog number, clone number, and/**OR** RRID.	Yes	Materials and Methods
**Primary cultures:** Provide species, strain, sex of origin, genetic modification status.	Yes	Materials and Methods
Report if the cell lines were recently **authenticated** (e.g., by STR profiling) and tested for mycoplasma contamination.	Not Applicable	

Experimental animals	Information included in the manuscript?	In which section is the information available? (Reagents and Tools Table, Materials and Methods, Figures, Data Availability Section)
**Laboratory animals or Model organisms:** Provide species, strain, sex, age, genetic modification status. Provide accession number in repository **OR** supplier name, catalog number, clone number, **OR** RRID.	Yes	Materials and Methods
**Animal observed in or captured from the field:** Provide species, sex, and age where possible.	Not Applicable	
Please detail **housing and husbandry conditions**.	Yes	Materials and Methods

Plants and microbes	Information included in the manuscript?	In which section is the information available? (Reagents and Tools Table, Materials and Methods, Figures, Data Availability Section)
**Plants:** provide species and strain, ecotype and cultivar where relevant, unique accession number if available, and source (including location for collected wild specimens).	Not Applicable	
**Microbes:** provide species and strain, unique accession number if available, and source.	Not Applicable	

Human research participants	Information included in the manuscript?	In which section is the information available? (Reagents and Tools Table, Materials and Methods, Figures, Data Availability Section)
If collected and within the bounds of privacy constraints report on age, sex and gender or ethnicity for all study participants.	Not Applicable	

Core facilities	Information included in the manuscript?	In which section is the information available? (Reagents and Tools Table, Materials and Methods, Figures, Data Availability Section)
If your work benefited from core facilities, was their service mentioned in the acknowledgments section?	Yes	Acknowledgements

Design

Study protocol	Information included in the manuscript?	In which section is the information available? (Reagents and Tools Table, Materials and Methods, Figures, Data Availability Section)
If study protocol has been **pre-registered, provide DOI in the manuscript**. For clinical trials, provide the trial registration number **OR** cite DOI.	Not Applicable	
Report the **clinical trial registration number** (at ClinicalTrials.gov or equivalent), where applicable.	Not Applicable	

Laboratory protocol	Information included in the manuscript?	In which section is the information available? (Reagents and Tools Table, Materials and Methods, Figures, Data Availability Section)
Provide DOI OR other citation details if **external detailed step-by-step protocols** are available.	Yes	Data analysis code in Data availability section: https://github.com/biocorecrg/allele_specific_RNAseq

Experimental study design and statistics	Information included in the manuscript?	In which section is the information available? (Reagents and Tools Table, Materials and Methods, Figures, Data Availability Section)
Include a statement about **sample size** estimate even if no statistical methods were used.	Not Applicable	Sample size was not determined by calculations.
Were any steps taken to minimize the effects of subjective bias when allocating animals/samples to treatment (e.g. **randomization procedure**)? If yes, have they been described?	Not Applicable	Not applicable
Include a statement about **blinding** even if no blinding was done.	Not Applicable	Not applicable
Describe **inclusion/exclusion criteria** if samples or animals were excluded from the analysis. Were the criteria pre-established?<br><br>If sample or data points were omitted from analysis, report if this was due to attrition or intentional exclusion and provide justification.	Not Applicable	No samples were excluded from the analysis.
For every figure, are **statistical tests** justified as appropriate? Do the data meet the assumptions of the tests (e.g., normal distribution)? Describe any methods used to assess it. Is there an estimate of variation within each group of data? Is the variance similar between the groups that are being statistically compared?	Yes	Statistical tests used were mentioned in the Figure Legends. Normal distribution was assessed to justify statistical tests.

Sample definition and in-laboratory replication	Information included in the manuscript?	In which section is the information available? (Reagents and Tools Table, Materials and Methods, Figures, Data Availability Section)
In the figure legends: state number of times the experiment was **replicated** in laboratory.	Yes	Figure Legends
In the figure legends: define whether data describe **technical or biological replicates**.	Yes	Figure Legends

Ethics

Ethics	Information included in the manuscript?	In which section is the information available? (Reagents and Tools Table, Materials and Methods, Figures, Data Availability Section)
Studies involving **human participants**: State details of **authority granting ethics approval** (IRB or equivalent committee(s), provide reference number for approval.	Not Applicable	
Studies involving **human participants**: Include a statement confirming that **informed consent** was obtained from all subjects and that the experiments conformed to the principles set out in the WMA Declaration of Helsinki and the Department of Health and Human Services Belmont Report.	Not Applicable	
Studies involving **human participants:** For publication of **patient photos**, include a statement confirming that consent to publish was obtained.	Not Applicable	
Studies involving experimental **animals**: State details of **authority granting ethics approval** (IRB or equivalent committee(s), provide reference number for approval. Include a statement of compliance with ethical regulations.	Yes	Materials and Methods
Studies involving **specimen and field samples:** State if relevant **permits** obtained, provide details of authority approving study; if none were required, explain why.	Not Applicable	

Dual Use Research of Concern (DURC)	Information included in the manuscript?	In which section is the information available? (Reagents and Tools Table, Materials and Methods, Figures, Data Availability Section)
Could your study fall under dual use research restrictions? Please check biosecurity documents and list of **select agents and toxins** (CDC): https://www.selectagents.gov/sat/list.htm	Not Applicable	
If you used a select agent, is the security level of the lab appropriate and reported in the manuscript?	Not Applicable	
If a study is subject to dual use research of concern regulations, is the name of the **authority granting approval and reference number** for the regulatory approval provided in the manuscript?	Not Applicable	

Reporting

The MDAR framework recommends adoption of discipline-specific guidelines, established and endorsed through community initiatives. Journals have their own policy about requiring specific guidelines and recommendations to complement MDAR.

Adherence to community standards	Information included in the manuscript?	In which section is the information available? (Reagents and Tools Table, Materials and Methods, Figures, Data Availability Section)
State if relevant guidelines or checklists (e.g., **ICMJE, MIBBI, ARRIVE, PRISMA**) have been followed or provided.	Not Applicable	
For **tumor marker prognostic studies,** we recommend that you follow the **REMARK** reporting guidelines (see link list at top right). See author guidelines, under 'Reporting Guidelines'. Please confirm you have followed these guidelines.	Not Applicable	
For **phase II and III randomized controlled trials**, please refer to the **CONSORT** flow diagram (see link list at top right) and submit the CONSORT checklist (see link list at top right) with your submission. See author guidelines, under 'Reporting Guidelines'. Please confirm you have submitted this list.	Not Applicable	

Data Availability

Data availability	Information included in the manuscript?	In which section is the information available? (Reagents and Tools Table, Materials and Methods, Figures, Data Availability Section)
Have **primary datasets** been deposited according to the journal's guidelines (see 'Data Deposition' section) and the respective accession numbers provided in the Data Availability Section?	Yes	Data availability Section. The accession number for the sequencing datasets reported in this paper is GEO: GSE169201 (https://www.ncbi.nlm.nih.gov/geo/query/acc.cgi?acc=GSE169201)
Were **human clinical and genomic datasets** deposited in a public access-controlled repository in accordance to ethical obligations to the patients and to the applicable consent agreement?	Not Applicable	
Are **computational models** that are central and integral to a study available without restrictions in a machine-readable form? Were the relevant accession numbers or links provided?	Not Applicable	
If publicly available data were reused, provide the respective **data citations in the reference list**.	Yes	References
